# Supplementary material for: Causal effect of video gaming on mental well-being in Japan 2020–2022
Source: Nat Hum Behav. 2024 Aug 19;8(10):1943–56. doi: 10.1038/s41562-024-01948-y (PMC11493677; doi:10.1038/s41562-024-01948-y)
Supplement: Supplementary file 1 — Supplementary methods, results, Tables 1–17, Figs. 1–19 and references. [file 41562_2024_1948_MOESM1_ESM.pdf]

nature human behaviour

Supplementary information https://doi.org/10.1038/s41562-024-01948-y

Causal effect of video gaming on mental well-being in Japan 2020–2022

In the format provided by the authors and unedited

Supplementary Information

Title: Causal effect of video gaming on mental well-being in Japan 2020-2022

Contents

Supplementary Methods.
• Method 1. Additional information on lottery, sampling process, and data collection.
► Method 1.1. Lotteries for Nintendo Switch and PlayStation5.
► Method 1.2. Difference between Nintendo Switch and PlayStation5.
► Method 1.3. Sampling process.
► Method 1.4. Lottery questionnaire.
► Method 1.5. Omnibus online survey.
• Method 2. Additional details on statistical analysis.
► Method 2.1. Causal assumptions.
► Method 2.2. Reduced form analysis (multivariate regression).
► Method 2.3. Pre-analysis: examining correlation with multivariate regression.
► Method 2.4. Propensity score matching design choices.
► Method 2.5. Instrumental variable approach.
► Method 2.6. Machine learning (causal forest) details.
► Method 2.7. Pseudo outcome tests.
► Method 2.8. Assessment of conditional unconfoundedness assumption.
➳ Method 2.8.1. Additional analysis 1: Imputation Approach for Game-Console-Purchase-Motivation.
➳ Method 2.8.2. Additional analysis 2: Short-period subsample analysis.
► Method 2.9. Causal assumptions and reliability in instrumental variable causal forest estimation.

Supplementary Results.
• Result 1. Preliminary analysis (association analysis) results.
• Result 2. Multivariate regression results of MSPD and SPD dummy variables.
• Result 3. Balance tables.
• Result 4. Assessment of balance and common support in propensity score matching analysis.
• Result 5. Assessment of conditional unconfoundedness assumption for ITT analysis.
► Result 5.1. Additional analysis 1: Imputation Approach for Game-Console-Purchase-Motivation.
► Result 5.2. Additional analysis 2: Short-period subsample analysis.
► Result 5.3. Causal diagram considerations.
► Result 5.4. Reflecting on causal diagrams with multiple lottery rounds.
• Result 6. Sensitivity checks with alternative model selections.

• Result 7. Subgroup analysis and quantile treatment effect analysis using instrumental variable method.
• Result 8. Machine learning taking SWLS as the outcome.
• Result 9. Examination of confounding in instrumental variable causal forest estimation.

Supplementary Tables.

• Supplementary Table 1: Video gaming preference and video game play time.
• Supplementary Table 2: Correlation between game engagement and mental well-being.
• Supplementary Table 3: Baseline characteristics' balance table for PS5 lottery winners and non-winners.
• Supplementary Table 4: Baseline characteristics' balance table for Nintendo Switch lottery winners and non-winners.
• Supplementary Table 5: Assessing unconfoundedness for game console lottery data: pseudo outcomes.
• Supplementary Table 6: Alternative outcome variables examined by multivariate regressions: MSPD and SPD.
• Supplementary Table 7: Comparison of baseline characteristics between respondents and nonrespondents.
• Supplementary Table 8: Causal impact of video game engagement on well-being in Japan.
• Supplementary Table 9: Weak instrument tests.
• Supplementary Table 10: First-stage regression results of instrumental variable method.
• Supplementary Table 11: Major theories, mechanisms, and hypotheses in literature.
• Supplementary Table 12: Number of observations for each round.
• Supplementary Table 13: Exposures: variables capturing game engagement.
• Supplementary Table 14: Outcomes and exposures collected in each round.
• Supplementary Table 15: Description of covariates.
• Supplementary Table 16: List of covariates used as features in causal forest algorithm.
• Supplementary Table 17: Outcomes by exposure.

Supplementary Figures.

• Supplementary Figure 1: Causal effect of winning game console lotteries on gaming time (N=3,491).
• Supplementary Figure 2: Balance check before and after propensity score matching for estimations regarding PlayStation5.
• Supplementary Figure 3: Balance check before and after propensity score matching for estimations regarding Nintendo Switch.
• Supplementary Figure 4: Common support check for propensity score matching estimations regarding PlayStation5.
• Supplementary Figure 5: Common support check for propensity score matching estimations regarding Nintendo Switch.
• Supplementary Figure 6: Intention-to-Treat estimates using Imputed-Console-Purchase-Motivation instead of the number of times of lottery participation (N=8,192).
• Supplementary Figure 7: Intention-to-Treat estimates using subsample of short period data.

• Supplementary Figure 8: Causal diagrams representing Intention-to-Treat analysis.

• Supplementary Figure 9: Causal diagram with two ‘turns’ representing Intention-to-Treat analysis.

• Supplementary Figure 10: Sensitivity checks for estimating impact of winning game console lottery on well-being (N=8,192).

• Supplementary Figure 11: Causal effect of ownership of PS5 on gaming time estimated by instrumental variable method (N=3,491).

• Supplementary Figure 12: Subgroup analysis of treatment effect using instrumental variable method.

• Supplementary Figure 13: Quantile treatment effect analysis.

• Supplementary Figure 14: Effect modification of video game ownership by gaming preference (N=8,192).

• Supplementary Figure 15: Survey participants and analysis sample.

• Supplementary Figure 16: Survey schedule and video game consoles’ lotteries.

• Supplementary Figure 17: Price history of video game consoles (Japanese Yen).

• Supplementary Figure 18: Machine learning results of effect modification in PlayStation5 ownership effect on life satisfaction (N=6,419).

• Supplementary Figure 19: Comparison of estimates between instrumental variable regression and instrumental variable causal forest on the impact of video game console possession on well-being in Japan (N=8,192).

Supplementary References.

Supplementary Methods.

Method 1: Additional information on lottery, sampling process, and data collection.

Method 1.1. Lotteries for Nintendo Switch and PlayStation5.

Since their initial releases in 2017 and 2020, the Nintendo Switch and PlayStation 5 (PS5) have achieved widespread global recognition. By 2022, global sales figures had reached 100 million units for the Nintendo Switch and 10 million for the PS5. The COVID-19 pandemic further boosted the number of players, with these consoles serving as online communication platforms for connecting with friends and family.[1]

The limited supply of semiconductors has significantly hindered the general public in Japan from purchasing the Nintendo Switch and PS5 through standard channels, such as online or in-store shopping. Instead, most consumers have had to resort to participating in lotteries managed by retailers to acquire the consoles. Alternatively, one could explore the secondary market to acquire the gaming consoles, albeit at an additional cost. While the supply-side challenge for PS5 persists, the issue with the Nintendo Switch was resolved by early 2021. See Supplementary Figure 17 for the price history of the Nintendo Switch and PS5.

It is essential to emphasize that retailers refrained from utilizing private information, such as gender or age, in their customer targeting. The participants were required to provide personal information, including their name and email address, via the retailer's website or in person. Once an individual wins a lottery, they gain the privilege of purchasing a gaming console. Although certain retailers mandate membership in their clubs for lottery participation, membership is accessible to everyone. Those facts support the unconfoundedness of the lottery result.

Contrary to a potentially simplified view of the process—wherein an individual participates in a lottery, awaits the result, and decides on further participation based on the outcome—the reality is somewhat more complex. Participants have the option to enter multiple lotteries concurrently, often with assistance from family members. For example, an individual intensely desiring a game

console may participate in lotteries from various retailers while also involving family members—an aspect accounted for in our data (our variable representing the number of lottery participation includes those of family members).

Furthermore, the announcement of lottery results does not follow a consistent timeline, with the waiting period fluctuating between two weeks to several months. Retailers also lack a uniform schedule for launching new lotteries. Consequently, non-winning participants cannot instantly join the next lottery and must wait until new ones are announced. Additionally, participants are free to enter new lotteries without waiting for the results of previous ones.

Method 1.2. Difference between Nintendo Switch and PlayStation5.

The Nintendo Switch and PS5 differ in several ways that affect their causal effects on well-being.[2,3] Firstly, the PS5 requires a TV connection for gameplay, whereas the Nintendo Switch can be played both with a TV and as a portable device anywhere, enabling outdoor play with others. This makes the Nintendo Switch more likely to be used for in-person play with family or friends, while PS5 users typically play alone. Secondly, the Nintendo Switch is popular among casual gamers and offers many exergames, while the PS5 is primarily targeted at hardcore gamers and includes fewer exergames. The pivotal role of the game genre in influencing well-being is well-supported by existing research.[3]

Method 1.3. Sampling process.

The survey sample size, as shown in Supplementary Table 12, varied each month based on the business objective of gameage R&I (GRI), the research firm conducting the survey. GRI aims to collect a specified number of opinions from individuals who use particular gaming software each month, with the goal of selling this information to its customers, namely, video game companies. Thus, if the number of participants using the gaming software is low, GRI will need to gather more

responses that month. Conversely, if more participants use the software in a given month, GRI may decrease the sample size for that period. Namely, GRI used no statistical methods to pre-determine sample sizes.

Initially, GRI uses stratified random sampling (stratified by gender, age, and gaming preference) for collecting the data. Yet, the number of observations for each stratum is not always equivalent. Because GRI's business purpose is to gather information on video games, they tend to collect more samples from people who enjoy video games. Consequently, our data tends to have smaller samples for older individuals.

Participants were blind during the survey. The survey agency (Cross Marketing) removed specific responses, including those with too many missing answers, among other low-quality indicators—a process to which the researchers were blind. Upon initiating data analysis, the researchers were unblinded. Although the survey agency compensated the participants, the exact details of this payment were not disclosed to the research team.

Method 1.4. *Lottery questionnaire.*

We gathered data regarding the Nintendo Switch/PS5 lotteries through two question types. Firstly, we asked respondents about the time that they, their household members, or relatives joined the lotteries to purchase the video game consoles for their households, respectively (separately for the Nintendo Switch/PS5). We then summed these entries (for each respondent) and used them to indicate the number of times respondents joined the lotteries. Secondly, we inquired about the retailers (for example, Amazon, Yodobashi Camera, and Sony Store) where the members (they/their household members/relatives) joined the lotteries and the number of times for each. We computed the total number of times the household participated in the lotteries by adding up the individual numbers for each retailer. As anticipated, the two questions yielded similar results for the total number of lottery participations. These questions aimed to help respondents recall their lottery

participation as accurately as possible. Our analysis used the first item, and the results were not affected by using the second item. Some respondents indicated they had not yet received lottery results, and we included them as having “not win lottery=lose lottery.”

Method 1.5. Omnibus online survey.

Gameage R&I (GRI), a gaming market research firm, conducts regular monthly surveys of individuals who have pre-registered through the survey agency Cross Marketing. The purpose of these surveys is to gather consumer data specifically related to the video game industry. Additional questions of ours were incorporated throughout five rounds of surveys conducted in December 2020, March, May, November 2021, and March 2022.

Our data constitutes a blend of panel and repeated cross-sectional observations. For example, 35.90% (22,091/61,523) of the respondents answered our survey more than once; 1.25% (771/61,523) answered five times. This data structure facilitates robustness checks, including placebo analysis.

Method 2: Additional details on statistical analysis.

Method 2.1. Causal assumptions.

The causal inference method and the ATE (average treatment effect) estimand used in this study is based on the potential outcomes framework. When using methods such as multivariate regression or propensity score matching (PSM), it is essential to consider the underlying assumptions for the estimates to be interpreted as causal. Specifically, three key assumptions must hold: conditional exchangeability (also known as conditional unconfoundedness, conditional mean independence, or unconfoundedness), positivity, and consistency.[4] Conditional exchangeability implies that, after adjusting measured covariates, treatment assignment is independent of the potential outcomes. Positivity implies that there is a non-zero conditional probability of receiving treatment for all observations. Finally, consistency implies that the definition of treatment is consistent for all observations.

Conditional exchangeability can be expressed as follows:

$$(Y_{0i}, Y_{1i}) \perp D_i \mid X_i$$

where Y_{0i} and Y_{1i} represent the potential outcomes for a treatment variable D_i and X_i is a set of observed covariates. Similarly, for the PSM method using the propensity score $\pi(X_i)$, conditional exchangeability is expressed as $(Y_{0i}, Y_{1i}) \perp D_i \mid \pi(X_i)$.

Conditional exchangeability is ultimately untestable. However, a natural experimental design enhances the plausibility of conditional exchangeability. To ensure this plausibility, it is important to examine the balance of covariates between the treatment and control groups, which can be found in the balance tables (Supplementary Table 3 and Supplementary Table 4). Due to the advantages of the natural experimental setting in game console lotteries, covariates are well-balanced. Furthermore, as recommended in the literature,[5] we also used pseudo outcome tests to ensure that unconfoundedness holds (Supplementary Table 5).

In our multivariate regression approach, it is important to adjust for the number of times respondents (and their household members) participated in the lotteries as X_i. Therefore, we assume that the results of the game console lotteries are independent, given the number of times respondents participated.

Positivity is ensured through the common support check graphs (Supplementary Figure 4 and Supplementary Figure 5). Due to the natural experimental setting, there is a good overlap between the control and exposed populations.

Finally, it is plausible to assume consistency because the treatment is well-defined. The two types of treatments, winning a Nintendo Switch lottery and winning a PlayStation5 lottery, are clearly defined. Moreover, when using the instrumental variable method, treatment (exposure) variables such as possession of Nintendo Switch, possession of PlayStation5, played Nintendo Switch last month, played PlayStation5 last month, and video gaming time are also well-defined.

In the propensity score matching approach, we additionally test the balance after matching, in order to validate the assumption of conditional exchangeability. The balance after matching can be found in Supplementary Figure 2 and Supplementary Figure 3, and the results indicate an improvement in the balance. This strengthens confidence in the validity of the conditional exchangeability assumption.

The instrumental variable method is frequently used to estimate the local average treatment effect (LATE) in a randomized controlled trial when imperfect compliance is present. In our case, the treatment group—lottery winners—cannot be compelled to purchase a Nintendo Switch or PlayStation5, resulting in imperfect compliance. Our instrument is winning Nintendo Switch/PlayStation5 lotteries, and our exposure variables (or endogenous variable) are game engagement variables, such as possession of Nintendo Switch/PlayStation5, playing Nintendo Switch/PlayStation5, and video gaming time.

To interpret the LATE estimated by the instrumental variable method as causal, there are several key assumptions; monotonicity, independence of the instrument, exclusion restriction, and no weak instrument assumption. Firstly, monotonicity implies that the instrument moves all participants' decisions of getting treatment (for example, possession of Nintendo Switch/PlayStation5) in the same direction. Secondly, the independence of the instrument implies that the instrument is uncorrelated with the error term. Third, the exclusion restriction states that the instrument only affects the outcome through its influence on the exposure variable, meaning no alternative pathways or channels link the instrument and the outcome. Lastly, the weak instrument assumption emphasizes the necessity of a strong enough correlation between the instrument and the exposure variable to yield precise and reliable effect estimates.

In our study, firstly, it is reasonable to assume that monotonicity holds since winning a lottery does not result in an individual deciding not to purchase a Nintendo Switch/PlayStation5. Secondly, the plausibility of the independence of the instrument, which is the outcome of a game console lottery, has been established in the preceding paragraphs. Thirdly, the weak instrument assumption has been tested through weak instrument tests, and no problems have been identified (Supplementary Table 9).

Finally, while direct testing of the exclusion restriction is not possible, we find it plausible to assume that possession of a Nintendo Switch and possession of a PlayStation5 satisfy the exclusion restriction. For example, it is challenging to envision a pathway where winning a Nintendo Switch lottery impacts the well-being of the winner without involving possession of a Nintendo Switch. However, when considering other exposure variables, such as playing Nintendo Switch last month or video gaming time, the existence of alternative channels cannot be ruled out entirely. For instance, an individual who acquires a Nintendo Switch may end up spending more time online, which could impact their well-being. Additionally, Nintendo Switch and PlayStation5 offer functionalities such as online video streaming (exemplified by platforms like YouTube) and Blu-ray playback (exclusive to

PlayStation5), though these are not presumed to be the primary utilities sought by users of these gaming consoles. Despite the potential for exclusion restriction violations, we believe it is valuable to incorporate a range of exposure variables. Notably, video gaming time is a worthy topic of discussion since it allows us to explore optimal gaming time within a causal inference framework.

In addition to OLS linear regression models, PSM, and instrumental variable methods, we employed instrumental variable causal forests. The estimates drawn by this technique also need assumptions to be considered causal. Further details are discussed in Method 2.6 and 2.9.

Method 2.2. Reduced form analysis (multivariate regression).

To conduct an Intention-to-Treat (ITT) analysis (or a reduced form analysis), we use the following equation:

$$Y_{ip} = \beta lottery_{ip} + \psi X_{ip} + \phi join_{ip} + \alpha_p + \epsilon_{ip} \quad (1)$$

where Y_{ip} is an outcome of interest such as well-being measures for an individual i in prefecture p. $lottery_{ip}$ is a dummy variable taking 1 if an individual wins the lotteries and 0 otherwise. The coefficient β is the parameter of interest. X_{ip} is a set of control variables consisting of individual characteristics, including age, gender, marital status, job status, and whether having children or not. In addition, we include gaming preference as control variables: dummy variables of each category (hardcore gamer, core gamer, middle-core gamer, casual gamer, and non-gamer). $join_{ip}$ is the number of times of joining the lotteries for the household of individual i. α_p are prefecture dummy variables.

We also use the following specification:

$$Playtime_{ip} = \beta lottery_{ip} + \psi X_{ip} + \phi join_{ip} + \alpha_p + \epsilon_{ip} \quad (2)$$

where $Playtime_{ip}$ is video game play time for individual i in prefecture p. $lottery_{ip}$ is a dummy variable taking 1 if an individual wins the lotteries and 0 otherwise. The coefficient β is the parameter of interest. We control for a set of control variables explained before. It is worth noting

that our primary analysis targeted only those who participated in the video game console lottery (n = 1,773 for Nintendo Switch, 6,419 for PS5).

Method 2.3. Pre-analysis: examining correlation with multivariate regression.

We use the following specification:

$$Y_{ip} = \beta Game_{ip} + \zeta X_{ip} + \alpha_p + \epsilon_{ip} \quad (3)$$

where $Game_{ip}$ is a measure of video game engagement for individual i in prefecture p. The rest of the variables are as defined above.

Method 2.4. Propensity score matching design choices.

We implemented the propensity score matching approach on our data based on Imbens (2015).[5] In addition to the summary outlined in Table 2, we present the specifics of our design decisions.

Logit models were utilized in this study. Specifically, the STATA command psmatch2 was used to conduct the matching process. To select the variables for the matching process, the STATA command psestimate, which follows Imbens (2015),[5] was used. The selected variables, including the second-order terms, can be found in figures Supplementary Figure 2 and Supplementary Figure 3. This approach was chosen to evade any arbitrary selection of variables.

To further improve the quality of the matching process, observations with propensity scores less than 0.1 and more than 0.9 were trimmed, as recommended by Imbens (2015).[5] The propensity score for trimming purposes was estimated using a design that involved one-to-one nearest neighbor matching without replacement and in descending order.

After applying trimming to the data, propensity score matching analysis was conducted to estimate ATE (or more precisely, ITT). To achieve improved precision, a design that involved two-

to-one nearest neighbor matching with replacement was employed, following the recommendation in Imbens (2015)[5] and Austin (2010).[6]

Overall, the study's methodological approach ensured that the differences in the outcomes between the treatment and control groups were solely attributed to the treatment effect. Moreover, the ITT assessed through the procedure above has been demonstrated to be robust with various other design choices in PSM (see Supplementary Result 6 and Supplementary Figure 10).

Method 2.5. Instrumental variable approach.

We examined various treatment variables, also known as exposure variables (or endogenous variables) in Figure 2. We acknowledge that possession of a PlayStation5 or Nintendo Switch (respectively for each estimation) yields the most reliable causal inference, given that the exclusion constraint is plausibly unbreached. Owning a game console is the most fundamental process for receiving benefits or drawbacks after winning a game console lottery. On the other hand, the remaining variables are not free from exclusion restriction issues. For instance, winning a PS5 lottery may not solely affect mental well-being through video gaming, as prolonged web browsing due to purchasing a PS5 may also have an impact. Therefore, using gaming time or playing experience of the last month as a treatment variable may violate the exclusion restriction.

Despite the potential exclusion restriction violation, we present multiple instrumental variable models with various treatment variables because they provide useful interpretation. For instance, utilizing video gaming time as a treatment variable allows us to infer the effects on a person's mental well-being as video gaming time increases.

Method 2.6. Machine learning (causal forest) details.

We used the instrumental variable causal forest.[7,8] We predicted conditional LATEs (CLATEs, conditional local average treatment effects) (given treatment W_i and instrument Z_i,

treatment effect $\tau(x)$ is identified via $\tau(x) = Cov[Y_i, Z_i|X_i = x]/Cov[W_i, Z_i|X_i = x]$) capturing the causal effect of ownership of video game consoles (Nintendo Switch and PlayStation5, respectively for each estimation). For the identification of treatment effects, a set of assumptions for the instrumental variable approach must hold (discussed in Supplementary Method 2.9). Note that given a conditional homogeneity assumption, the CLATE is simply the conditional ATE (CATE; $\tau(x) = E(Y_{1i} - Y_{0i}|X_i = x)$).

The instrumental variable causal forest was implemented using the R package "grf", version 2.3.2. Predicted conditional average treatment effects were generated with selected parameters as follows: (i) the minimum leaf size was set to five (the default of the package), (ii) the number of trees in the forest was set to 10,000 (larger than the default: 2,000), (iii) the fraction of bootstrapped subsample used to build each tree was set to 50% (the default of the package), (iv) the number of covariates considered at each split was set to $\frac{K}{3}$, with K being the total number of predictors, following the empirical literature.[9,10] To ensure robust results that were not dependent on a particular sample and to reduce bias, we executed 1,000 simulations on different test samples (each employing a distinct 50-50 split of the data) and aggregated the results (i.e., bagging), following practices suggested by previous studies.[9,11] The list of covariates can be found in Supplementary Table 16.

Method 2.7. Pseudo outcome tests.

Following Imbens (2015),[5] we performed pseudo outcome tests (also known as placebo analyses), by utilizing pre-lottery values for K6 and SWLS as pseudo outcomes. For this, we utilized K6 and SWLS data from the second round—the initial round in which information was gathered regarding PS5 lotteries—and ran regressions (or applied the PSM method) using data from rounds 3-5. We conducted placebo analyses only for PS5 lotteries since pre-lottery outcomes were not available for Nintendo Switch lotteries. It is noteworthy that individuals who had already won PS5

lotteries in round 2 were removed from the placebo analysis samples. Otherwise, the outcomes of the people who had won lotteries were included as pseudo outcomes.

Method 2.8. Assessment of conditional unconfoundedness assumption.

A concern arises regarding the assumption of conditional ignorability; controlling solely for the number of lottery participations may not be sufficient. In the control group, we can directly observe the total number of participations without a win. However, the data for the treatment group is limited after an individual wins, as they stop participating. Imagine an individual who wins on the second attempt but would have continued participating; compare this with another who stops after two unsuccessful attempts. These participants are not statistically identical, casting doubt on the conditional ignorability assumption and necessitating further analysis to understand how significantly this caveat might impact our primary findings.

In this supplementary section, we attempt to alleviate the concern regarding the number of non-winning lottery participations, hereafter termed "Game-Console-Purchase-Motivation." This variable represents an individual's persistent effort and desire to acquire a game console through continuous lottery participation without winning. We acknowledge that we cannot directly observe the Game-Console-Purchase-Motivation for lottery winners (treatment group). However, for non-winners (control group), this variable aligns directly with their observed number of lottery participations. We use three strategies: (i) an imputation approach, (ii) analyses of short-period subsamples, and (iii) the application of causal diagrams for consideration of covariates.

Method 2.8.1 Additional analysis 1: Imputation Approach for Game-Console-Purchase-Motivation.

The first approach involved predicting Game-Console-Purchase-Motivation. Leveraging the control group's data, we developed a model to predict the Game-Console-Purchase-Motivation for

individuals within the treatment group. This issue closely aligns with the attrition problem, leading us to approach it through imputation. It is worth noting that out of 8,192 respondents, 2,323 were successful in the lottery. Using the data from the remaining 5,869 respondents, we imputed Game-Console-Purchase-Motivation values for the lottery winners, addressing the 28.3% (2,323/8,192) missing values.

The prediction utilized various individual characteristics, including gender, age, employment status, and gaming preferences. Given its ensemble learning capabilities and robustness, we opted for the random forest model, drawing from previous studies.[12] The process unfolded: We initiated a random forest model based on non-winning participants, then predicted Game-Console-Purchase-Motivation for lottery winners. Following the prediction phase, we established a new variable to represent Game-Console-Purchase-Motivation, replacing the original participation counts of lottery winners with the predicted values. This new variable— "Imputed-Console-Purchase-Motivation" — incorporates observed and predicted values.

Subsequently, using Imputed-Console-Purchase-Motivation instead of the actual participation counts, we developed alternative models. The models mirror our primary Intent-To-Treat (ITT) models—multivariate regression and propensity score matching. This alteration facilitated a comparison between the primary and alternative model results, enabling an examination of how the potential confounder influences the outcomes.

Method 2.8.2 Additional analysis 2: Short-period subsample analysis.

Another method we used involves analyzing data from shorter time periods. When the data is collected over a short time, and participants cannot enter new lotteries after losing one, the issues we are concerned about decrease. In this scenario, the observed number of lottery participations is almost the same as the Game-Console-Purchase-Motivation, which helps reduce potential biases from not considering this factor.

However, for this method to work, we need to know both when a respondent entered a lottery and if they won, when that happened. Our data for the Nintendo Switch lottery shows when participants entered but not when they won, and unfortunately, our PlayStation5 data does not have either piece of information.

We utilized available Nintendo Switch data for additional analysis, under the assumption that participants ceased entering lotteries upon winning. The full sample encompasses nine months, from March to November 2020. Within this timeframe, we examined shorter intervals: November alone (one month), October through November (two months), September through November (three months), August through November (four months), July through November (five months), and June through November (six months).

Our primary ITT analysis, which includes multivariate regression and the PSM method, was applied to these shorter interval subsamples. However, for the subsamples of one month (N=352), two months (N=514), and three months (N=681), the PSM estimates turned out unreliable and thus not displayed. These specific subsamples presented propensity score distributions that were not only irregular but also volatile, markedly differing from the distribution observed in the full sample. This significant discrepancy highlights a concerning lack of consistency and comparability, casting doubt on the reliability of the PSM results obtained from these smaller subsamples.

Method 2.9. Causal assumptions and reliability in instrumental variable causal forest estimation.

The C(L)ATEs estimated by generalized random forests (GRF) can be considered causal under the standard unconfoundedness assumption,[13] formally expressed as $(Y_{0i}, Y_{1i}) \perp D_i \mid X_i$. This foundational assumption, central to our study, has been extensively discussed in Supplementary Method 2 (this section). Likewise, the assumption underlying instrumental forests[7,14] aligns with the traditional instrumental variable approach (discussed in Supplementary method 2.1 and 2.5), including conditional independence of the instrument.

Hence, the plausibility of assuming unconfoundedness for the variation introduced by our natural experiment—the lottery—is essential for interpreting the instrumental variable causal forest estimates as causal. While unconfoundedness is not testable, it is recommended to assess whether the assumption is plausible.[5] Accordingly, we assessed the unconfoundedness thoroughly from multiple perspectives (particularly, Supplementary Tables 3-5 and Supplementary Figure 10).

Regarding the reliability of our instrumental variable causal forest estimation, in Figure 3 (panels a and b), we compare the estimates obtained from instrumental forests with those derived from traditional instrumental variable regressions. The estimated CLATEs are distributed around the LATEs, supporting the reliability of our instrumental forest estimates. In Supplementary Result 9, we provide further information on a concise additional check on whether our instrumental forest estimates suffer from confounding.

Supplementary Results.

Result 1: Preliminary analysis (association analysis) results.

Before beginning our primary analysis, as a preliminary analysis, we first examined the correlation between video gaming and well-being. We used several measures of video game engagement: i) whether respondent households had a Nintendo Switch, ii) whether respondents played Nintendo Switch last month, iii) whether respondent households had a PlayStation5 (PS5), iv) whether respondents played PS5 last month, v) video game play time (including any video game consoles and computer games). We employed Equation 3 in Supplementary Methods.

In Supplementary Table 2, we showed estimates of three specifications (models): (1) without control variables, (2) with control variables except for gaming preference dummy variables, and (3) with a full set of control variables. Through the comparison of these estimates, we aim to elucidate the extent to which the inclusion or exclusion of specific control variables affects the observed correlation.

First, as anticipated, we observed statistically significant positive correlations between psychological distress level measured by K6 and video game engagement, although not consistently across all estimates. It is important to highlight that a higher K6 score indicates poorer mental health; thus, a positive correlation between K6 and video gaming implies a negative association between mental health and video gaming. However, the observed effect sizes were modest, raising questions about their practical significance. For instance, when adopting a threshold of 0.2 SD units as the smallest effect size of interest,[15] the observed effects appear non-meaningful. Moreover, consistent with previous literature,[16] models incorporating a larger set of control variables displayed a smaller negative correlation. Notably, when adjusting for gaming preference in the third model, the beta (β) coefficient estimates were substantially altered; three out of the five estimates from Model 3 were not statistically significant. The marked variability in estimates across different models suggests the potential unreliability of these estimates.

Second, the analysis revealed a consistent positive correlation between SWLS scores and measures of video game engagement (Supplementary Table 2). While these findings might initially seem counterintuitive given certain public perceptions around gaming, they align well with recent studies,[17,18] which found positive associations between video game participation and well-being. Nevertheless, it is essential to note that those association analyses may not provide insight into the causal relationship.

Result 2: Multivariate regression results of MSPD and SPD dummy variables.

We found that winning a Nintendo Switch/PS5 lottery had a statistically significant positive impact on the two types of psychological distress dummy variables (Supplementary Table 6). As for the MSPD (mild-to-serious psychological distress) dummy variable, winning a Nintendo Switch/PS5 lottery had a statistically significant positive impact. As for the SPD (serious psychological distress) dummy variable, winning a Nintendo Switch lottery gave a statistically significant positive impact, whereas winning a PS5 lottery did not have a significant impact. The results supported the robustness of our findings, though some of the estimates were not significant.

Result 3: Balance tables.

Supplementary Table 3 and Supplementary Table 4 present the summary statistics comparison for the covariates of lottery winners and non-winners. The first table shows the balance of PS5 lotteries, while the second shows the balance of Nintendo Switch lotteries. Following previous studies,[5] we showed standardized differences (or normalized differences) between winners and non-winners. The balance tables present minor differences. For example, in Supplementary Table 3, the differences in characteristics between winner and non-winners are small, with only one out of 30 standardized differences larger than 0.2—a standardized difference considered small.[19] Only one variable (the number of times entered lotteries) exceeded 0.2. Yet, due to its nature, this variable is expected to vary between winners and losers. This variable was included as a covariate to

control for potential bias. Considering that the cutoff of standardized differences for assessing the balance after using PSM is 0.1,[20] one can observe that the characteristics are well-balanced even before PSM is applied. This supports the unconfoundedness of the treatment effect (effect of winning a game console lottery), which is vital for interpreting the results of multivariate regression and instrumental variable method as causal.

Result 4: Assessment of balance and common support in propensity score matching analysis.

A crucial step in propensity score matching analysis is to assess the balance of covariates between treatment and control groups, as well as the existence of common support in the propensity score distribution. We conducted balance checks for all covariates included in the matching procedure and found no significant differences between the treated and control groups. Supplementary Figure 2 and Supplementary Figure 3, presenting standardized differences, show the balance checks before and after matching. Even before matching, the standardized differences were small; most of them were smaller than 0.1 in absolute value. Importantly, after matching, all the covariates' standardized differences were smaller than 0.1, a commonly used cutoff.[20] Additionally, we examined the common support of the propensity score distribution and observed no evidence of overlap issues (Supplementary Figure 4 and Supplementary Figure 5). These results indicate that our propensity score matching analysis was successful in achieving a balance between the treatment and control groups.

Result 5: Assessment of conditional unconfoundedness assumption for ITT analysis.

Result 5.1. Additional analysis 1: Imputation Approach for Game-Console-Purchase-Motivation.

We proceeded with our primary ITT analysis using Imputed-Console-Purchase-Motivation instead of actual participation counts, incorporating both multivariate regression and propensity score matching. Supplementary Figure 6, formatted similarly to Figure 1 in the main text, displays the results for easy comparison. Findings derived from models using Imputed-Console-Purchase-

Motivation as a covariate— 'alternative model' —closely mirrored those presented in Figure 1, underscoring the robustness of our results even after the introduction of Imputed-Console-Purchase-Motivation as a covariate.

Result 5.2. Additional analysis 2: Short-period subsample analysis.

While using smaller samples can lead to unstable estimates with larger errors, the results from these short timeframes (Supplementary Figure 7) were mostly similar to the full sample's estimates, except in the two-month timeframe. Interestingly, the estimates from the four-month period, which had less than half the number of samples as the full period, were still consistent with the full sample's estimates. Given these findings, we gently propose that our main results are still valid, and the omission of Game-Console-Purchase-Motivation might not have a significant impact on the results.

Result 5.3. Causal diagram considerations.

In this subsection, we evaluate whether it is more appropriate to control for Game-Console-Purchase-Motivation (GCPM) or the actual number of lottery participation in our study. This assessment is conducted using causal diagrams, with the backdoor criterion guiding the analysis.

Supplementary Figure 8 presents three distinct causal diagrams for consideration. The first diagram (1) outlines a typical study design that employs lotteries as a natural experiment, devoid of considerations related to GCPM. Upon reassessment, two alternative diagrams, labeled as (2) and (3), surface as plausible alternatives to the initial design.

Diagram (2) underscores the need to control for GCPM, as it is a crucial variable influencing the study outcome. In contrast, Diagram (3) indicates that simply controlling for the number of times a respondent participates in the lottery is adequate to satisfy the backdoor criterion. This approach is grounded on the observation that GCPM does not directly influence lottery success. Rather, a higher number of lottery participations increases the chances of winning. Hence, for the purpose of achieving conditional ignorability, it may not be necessary to control for GCPM.

Result 5.4. Reflecting on causal diagrams with multiple lottery rounds.

The concerns related to GCPM as a potential confounder prompt us to reconsider the causal diagram, especially considering the multiple rounds or "turns" of lottery participation. For clarification, a "turn" represents a distinct round of lottery participation at a particular retailer.

Supplementary Figure 9 presents a revised causal diagram incorporating two "turns" of participation. While this figure illustrates the concept with two turns, actual participation involved multiple, sometimes overlapping, turns. It is worth noting that Supplementary Figure 8 did not account for these multiple turns.

As previously outlined, participants could enter lotteries at various retailers at different times. Ideally, data collection (of lottery information, gaming behavior, and well-being) would occur separately for each "turn," allowing for comparisons within each distinct turn. Under this ideal approach, the concerns related to GCPM as a potential confounder would be addressed effectively, as the winning or losing of future lotteries would not influence the observed number of lottery participations per turn.

Additionally, the causal diagram in Supplementary Figure 9 suggests that controlling for the number of lottery participation might not be appropriate when analyzing the causal relationship with data that aggregates all turns—our ITT analysis. The control variable might inadvertently incorporate information only available post hoc, after lottery outcomes have been determined. As a result, some might suggest not using the number of lottery participations as a control, but rather using GCPM instead. However, as demonstrated in Supplementary Figure 6, the alternative models using Imputed-Console-Purchase-Motivation yielded results similar to our primary models, affirming their robustness.

Result 6: Sensitivity checks with alternative model selections.

In Supplementary Figure 10, we presented several estimates with alternative model selections of regression and Propensity Score Matching (PSM). Initially, we demonstrated the estimates of regression without any adjustments. The estimates were observed to be very similar to those with adjustment, which is our principal model. This supports the idea that there was no confounding of the treatment effect.

Next, we exhibited estimates using several alternative design choices of PSM, commonly used for estimating either Average Treatment Effect on Treated (ATT) or Average Treatment Effect (ATE). Some might advocate for estimating the ATT—or, more precisely, in our study, Intention-to-Treat (ITT) effect on the assigned treatment group—using PSM without replacement. Nonetheless, our primary focus was the ATE (or the ITT effect) due to its consistency with our regression that also aimed to estimate the ATE (or the ITT effect). As a sensitivity check, we estimated ATT and found that the estimates are comparable to ATE. We further assessed the ATE using an alternate PSM design, one-to-one matching with replacement. The estimates were in line with prior results, though, as anticipated, the standard errors increased.

Result 7: Subgroup analysis and quantile treatment effect analysis using instrumental variable method.

To investigate whether the impact of video gaming varies based on the time spent, we conducted a subgroup analysis using the instrumental variable method. Recall that the instrumental variable estimate, where the exposure variable is video gaming time, implied that longer gaming was causally beneficial for mental well-being (Figure 2). We aim to investigate whether such benefits decrease as gaming time becomes more prolonged.

Initially, we performed instrumental variable quantile analysis. This provides treatment effect estimates for the outcome variable's quantile. Panel a of Supplementary Figure 13 suggests that the point estimate of prolonged gaming time is 2.5 hours at maximum, and even with the 95 percent

confidence interval, it could be a maximum of four hours. These findings align with panels c and d of Figure 3. It is crucial to note that these findings do not allow us to assert the effects of someone who did not play video games at all but began to game extremely long, such as for ten hours. To make such inferences utilizing a natural experimental approach, one requires an intervention that induces a ten-hour increase in video gaming time.

Additionally, it is worth stressing that the quantile treatment effect analysis highlights heterogeneity among outcome variables rather than exposure variables. Our focus is on the treatment effect heterogeneity in gaming time, an exposure variable. Thus, conventional quantile treatment effect analysis does not provide us with the information we require.

We, therefore, performed a subgroup analysis using the instrumental variable approach by restricting our sample to subgroups with a bandwidth of 60 percent, selected to address weak instrumental variable problems (Supplementary Figure 12). We conducted instrumental variable estimation for five subgroups, with ranges of (i) 0.2 to 0.8 quantile (0.3 to 2.3 hours), (ii) 0.25 to 0.85 quantile (0.3 to 2.9 hours), (iii) 0.3 to 0.9 quantile (0.3 to 3.6 hours), (iv) 0.35 to 0.95 quantile (0.6 to 5.7 hours), and (v) 0.4 to 1.00 quantile (0.9 to 10 hours). All five group estimates exhibited no weak instrument problems, as indicated by the Kleibergen-Paap F statistic (presented in notes in Supplementary Figure 12). We used quantiles to ensure that each subgroup had sufficient samples to avoid weak instrument problems. We employed video gaming time as the exposure variable, the same as the one used in Figure 2. The outcome variables are psychological distress (K6) in panel a and life satisfaction (SWLS) in panel b.

Panels of Supplementary Figure 12 indicate that the benefits of video gaming diminish as the duration of gaming increases. For instance, panel a indicates that increasing video gaming time for an additional hour reduces psychological distress by 1.2 standard deviations for subgroup (ii)—0.25 to 0.85 quantile (equivalent to 0.3 to 2.9 hours of video gaming). In contrast, increasing video gaming time for an additional hour has little or no positive psychological effect for subgroup (v)—

0.4 to 1.00 quantile (equivalent to 0.9 to 10 hours of video gaming). Estimating the threshold at which video gaming benefits begin to depreciate is difficult. However, both panels a and b suggest that playing video games for three hours or more is less advantageous than playing for a shorter duration.

We did not discover any evidence of a threshold for gaming duration that adversely affects mental well-being. This may be because our quasi-“intervention” did not encourage users to have additional gameplay of more than four hours (Supplementary Figure 13).

Result 8: Machine learning taking SWLS as the outcome.

Our machine learning outcomes exhibit robustness upon scrutinizing SWLS (Satisfaction with life scale). Supplementary Figure 18 elucidates that the manifestation of effect modification witnessed in SWLS parallels that of K6, albeit with diminished prominence for certain instances.

Result 9: Examination of confounding in instrumental variable causal forest estimation.

Here, we provide an additional robustness check for assessing whether our instrumental forest estimates suffer from confounding. We compare the LATEs estimated by the traditional instrumental variable regressions and those estimated by instrumental forests.

GRF extend their utility beyond estimating subgroup treatment effects by also enabling the estimation of LATE across the sample.[21] The LATE estimated using the GRF is referred to as the Average Conditional Local Average Treatment Effect (ACLATE), reflecting the estimation methodology.[21] The GRF employ Augmented Inverse-Probability Weighting (AIPW) for this purpose.[21,22] In our study, we found that simply averaging the CLATEs across all samples produces a point estimate that closely mirrors the AIPW estimate while using the AIPW estimates is recommended.[21]

Supplementary Figure 19 compares the LATE estimates derived from instrumental variable regressions (Two-Stage Least Squares) and instrumental forests. To facilitate clear understanding,

we designed Supplementary Figure 19 to reflect the layout of Figure 1 of the main text. The consistency between estimates from instrumental forests and those obtained through instrumental variable regressions serves as another piece of supportive evidence for the reliability of our approach, particularly ensuring the plausibility of the unconfoundedness of the heterogeneous treatment effect estimates. Although this figure and Figure 3 (panels a and b) present similar information, the unique visual layout of Supplementary Figure 19 may clarify how closely the estimates from instrumental forests and instrumental variable regressions align.

Supplementary Tables.

Supplementary Table 1: Video gaming preference and video game play time.

Variable	Mean (SD)				
	Hardcore gamer (n=7064)	Core gamer (n=9516)	Middle-core gamer (n=10762)	Casual gamer (n=9521)	Non-gamer (n=5159)
Video game playtime (hour/day)	1.626 (2.277)	1.108 (1.844)	0.624 (1.366)	0.323 (0.976)	0.139 (0.735)
Smartphone game playtime (hour/day)	1.381 (2.075)	1.268 (1.951)	1.072 (1.666)	0.673 (1.342)	0.212 (0.886)

Notes. Observations of rounds 4 and 5 were used. Gameplay time was measured at each survey round. Video gaming preferences for rounds 4 and 5 were measured in November 2020 and 2021, respectively. Whereas this table associates the most recent gaming preferences with gaming time for each survey round, our analyses, including balance checks, utilized the oldest available data, going back to 2019, to avoid bad control issues.

Supplementary Table 2: Correlation between game engagement and mental well-being.

Variables	Number of observations	β coefficient (95% CI)		
		Model 1	Model 2	Model 3
Outcome variable: Psychological distress (K6)				
Have a Nintendo Switch	18,912	0.02 (-0.012 to 0.053)	0.058 (0.024 to 0.091)	-0.008 (-0.043 to 0.026)
	P-value	0.225	0.001	0.641
Played Nintendo Switch this month	18,912	0.072 (0.031 to 0.114)	0.085 (0.045 to 0.126)	0.003 (-0.226 to 0.269)
	P-value	<0.001	<0.001	0.863
Have a PS5	78,690	0.112 (0.057 to 0.166)	0.136 (0.079 to 0.192)	0.079 (0.024 to 0.133)
	P-value	<0.001	<0.001	0.005
Played PS5 this month	78,690	0.054 (-0.021 to 0.131)	0.065 (-0.012 to 0.143)	-0.017 (-0.096 to 0.061)
	P-value	0.156	0.1	0.654
Video game play time (hour/day)	42,022	0.036 (0.028 to 0.044)	0.025 (0.018 to 0.032)	0.013 (0.005 to 0.021)
	P-value	<0.001	<0.001	<0.001
Outcome variable: Life satisfaction (SWLS)				
Have a PS5	78,690	0.147 (0.079 to 0.214)	0.12 (0.061 to 0.18)	0.139 (0.08 to 0.197)
	P-value	<0.001	<0.001	<0.001
Played PS5 this month	78,690	0.213 (0.12 to 0.307)	0.226 (0.14 to 0.312)	0.252 (0.166 to 0.339)
	P-value	<0.001	<0.001	<0.001
Video game play time (hour/day)	42,022	-0.012 (-0.018 to -0.006)	0.007 (0.001 to 0.012)	0.01 (0.004 to 0.015)
	P-value	<0.001	0.007	<0.001

Notes. K6, Kessler psychological distress scale; SWLS, The Satisfaction with Life Scale; CI, confidence intervals. Model 1: Adjusted for prefecture dummy variables and round dummy variables only. Model 2: Additionally adjusted for a set of covariates (i.e., age, gender, marital status, employment status, whether having children or not, and occupation) in Supplementary Table 15. Model 3: Additionally adjusted for gaming preference dummy variables. Standard errors were clustered by prefectures. A lower K6 means having less psychological distress, while a higher SWLS means greater life satisfaction. The estimates were standardized by the standard deviations. The two-sided t-test was used as the statistical test. No adjustments for multiple comparisons were made.

Supplementary Table 3: Baseline characteristics' balance table for PS5 lottery winners and non-winners.

Variable	Did not win PS5 lottery (N=5022) Mean/SD	Won PS5 lottery (N=1397) Mean/SD	Normalized difference
# of times joined lottery	4.257 [6.309]	6.130 [8.188]	-0.275
Age	36.988 [15.724]	37.425 [15.454]	-0.028
Gender (Male)	0.619 [0.486]	0.598 [0.490]	0.042
Married	0.593 [0.491]	0.592 [0.492]	0.003
Divorced/separated	0.051 [0.220]	0.052 [0.223]	-0.005
Have child(ren)	0.511 [0.500]	0.516 [0.500]	-0.010
Student	0.212 [0.409]	0.199 [0.399]	0.032
Stay-at-home wife/husband	0.069 [0.253]	0.081 [0.273]	-0.047
Full-time employee	0.499 [0.500]	0.458 [0.498]	0.082
Part-time employee	0.088 [0.283]	0.112 [0.315]	-0.083
Self employed/others	0.063 [0.243]	0.069 [0.254]	-0.027
Unemployed/not a student	0.070 [0.255]	0.081 [0.273]	-0.042
Gaming Preference			
Hardcore gamer	0.362 [0.481]	0.349 [0.477]	0.028
Core gamer	0.254 [0.435]	0.249 [0.433]	0.012
Middle-core gamer	0.172 [0.378]	0.170 [0.376]	0.005
Casual gamer	0.089 [0.285]	0.105 [0.307]	-0.055
Non-gamer	0.122 [0.327]	0.127 [0.333]	-0.015
Job: industries			
Engineering and construction[1]	0.065 [0.246]	0.077 [0.267]	-0.049
Textile and Cosmetics[2]	0.070 [0.255]	0.060 [0.238]	0.039
Manufacturing	0.134 [0.340]	0.155 [0.362]	-0.062
Trading and Mass media[3]	0.037 [0.188]	0.030 [0.171]	0.037
Distributors, Retailers	0.046 [0.210]	0.038 [0.191]	0.040
Carriers[4]	0.045 [0.206]	0.041 [0.198]	0.019
Public works	0.054 [0.226]	0.062 [0.240]	-0.034
IT industries[5]	0.067 [0.250]	0.059 [0.235]	0.034
Banks and Financial services	0.036 [0.187]	0.026 [0.161]	0.055
Food and Other Services[6]	0.104 [0.305]	0.096 [0.295]	0.027
Medical care, Welfare	0.063 [0.243]	0.059 [0.236]	0.015
Education	0.035 [0.183]	0.033 [0.179]	0.009
Others[7]	0.067 [0.249]	0.066 [0.248]	0.003

Notes. PS5, PlayStation5. Respondents' characteristics are displayed. Caregivers' characteristics are used where appropriate.

[1]Civil engineering, Construction, Real estate, Housing and building services; [2]Daily necessaries, Textile and apparel, Cosmetics, Food and Beverages; [3]Trading companies, Publishing, Printing, Mass media; [4]Carriers, Warehousing, Logistics; [5]Software and Information services; [6]Food services, Hairdressing, Cosmetology, Other Services; [7]Other industries and types of business.

Supplementary Table 4: Baseline characteristics' balance table for Nintendo Switch lottery winners and non-winners.

Variable	Did not win Nintendo Switch lottery (N=847)	Won Nintendo Switch lottery (N=926)	Normalized difference
	Mean/SD	Mean/SD	
# of times joined lottery	3.336 [4.170]	4.449 [5.227]	-0.233
Age	35.967 [16.575]	36.254 [15.919]	-0.018
Gender (Male)	0.564 [0.496]	0.504 [0.500]	0.120
Married	0.643 [0.479]	0.692 [0.462]	-0.104
Divorced/separated	0.052 [0.222]	0.039 [0.193]	0.063
Have child(ren)	0.580 [0.494]	0.609 [0.488]	-0.060
Student	0.241 [0.428]	0.233 [0.423]	0.018
Stay-at-home wife/husband	0.084 [0.277]	0.102 [0.302]	-0.061
Full-time employee	0.442 [0.497]	0.455 [0.498]	-0.026
Part-time employee	0.096 [0.294]	0.098 [0.298]	-0.009
Self employed/others	0.074 [0.263]	0.058 [0.234]	0.065
Unemployed/not a student	0.064 [0.244]	0.054 [0.226]	0.042
Gaming Preference			
Hardcore gamer	0.359 [0.480]	0.344 [0.475]	0.030
Core gamer	0.188 [0.391]	0.200 [0.400]	-0.030
Middle-core gamer	0.172 [0.378]	0.216 [0.412]	-0.110
Casual gamer	0.109 [0.311]	0.100 [0.301]	0.027
Non-gamer	0.172 [0.378]	0.139 [0.346]	0.091
Job: industries			
Engineering and construction[1]	0.071 [0.257]	0.070 [0.256]	0.003
Textile and Cosmetics[2]	0.051 [0.220]	0.066 [0.248]	-0.064
Manufacturing	0.129 [0.335]	0.103 [0.304]	0.082
Trading and Mass media[3]	0.037 [0.188]	0.037 [0.188]	-0.001
Distributors, Retailers	0.045 [0.207]	0.044 [0.206]	0.003
Carriers[4]	0.052 [0.222]	0.032 [0.177]	0.098
Public works	0.059 [0.236]	0.051 [0.220]	0.036
IT industries[5]	0.055 [0.229]	0.070 [0.256]	-0.060
Banks and Financial services	0.034 [0.182]	0.036 [0.185]	-0.008
Food and Other Services[6]	0.105 [0.307]	0.103 [0.304]	0.008
Medical care, Welfare	0.071 [0.257]	0.070 [0.256]	0.003
Education	0.032 [0.176]	0.025 [0.156]	0.043
Others[7]	0.079 [0.270]	0.068 [0.252]	0.042

Notes. Respondents' characteristics are displayed. Caregivers' characteristics are used where appropriate.
[1]Civil engineering, Construction, Real estate, Housing and building services; [2]Daily necessaries, Textile and apparel, Cosmetics, Food and Beverages; [3]Trading companies, Publishing, Printing, Mass media; [4]Carriers, Warehousing, Logistics; [5]Software and Information services; [6]Food services, Hairdressing, Cosmetology, Other Services; [7]Other industries and types of business.

Supplementary Table 5: Assessing unconfoundedness for game console lottery data: pseudo outcomes.

Outcome variables	Pseudo Outcome (K6, Standardized) β coefficient (95% CI)		Pseudo Outcome (SWLS, Standardized) β coefficient (95% CI)	
	Model 1: Regression	Model 2: PSM	Model 1: Regression	Model 2: PSM
Win PS5 lottery	0.04 (-0.105 to 0.187)	0.069 (-0.11 to 0.248)	-0.002 (-0.189 to 0.185)	-0.004 (-0.16 to 0.151)
Number of observations	1,621	1,379	1,621	1,379

Notes. PS5, PlayStation5; K6, Kessler psychological distress scale; SWLS, The Satisfaction with Life Scale; PSM, Propensity Score Matching; CI, Confidence interval. Coefficients were standardized using standard deviation. Pseudo outcomes are K6 and SWLS measured at round 2. The sample consists of rounds 3-5 observations. Regressions were adjusted for a set of control variables (i.e., age, gender, marital status, employment status, whether having children or not, occupation, gaming preference, and the number of times joining game console lotteries) in Supplementary Table 15, prefecture dummies, and round dummies. Regression standard errors were clustered by prefectures. Abadie-Imbens robust standard errors were used for PSM.

Supplementary Table 6: Alternative outcome variables examined by multivariate regressions: MSPD and SPD.

	Mild-to-serious psychological distress (K6>=5)			Serious psychological distress (K6>=13)		
		β coefficient (95% CI)			β coefficient (95% CI)	
Variables	%	Model 1	Model 2	%	Model 1	Model 2
Participants who joined Nintendo Switch lottery (n=1,773)						
Win Switch lottery	60.0	-0.108 (-0.152 to -0.064)	-0.101 (-0.136 to -0.065)	29.1	-0.058 (-0.094 to -0.022)	-0.059 (-0.093 to -0.025)
	P-value	<0.001	<0.001		0.002	0.001
Participants who joined PlayStation5 lottery (n=6,419)						
Win PS5 lottery	54.8	-0.04 (-0.069 to -0.011)	-0.047 (-0.073 to -0.021)	22.8	-0.011 (-0.036 to 0.013)	-0.016 (-0.041 to 0.008)
	P-value	0.007	<0.001		0.362	0.192

Notes. Switch, Nintendo Switch; PS5, PlayStation5; CI, confidence intervals; MSPD, mild-to-serious psychological distress; SPD, serious psychological distress. Model 1: Adjusted for prefecture dummy variables and round dummy variables only. Model 2: Additionally adjusted for a set of covariates (i.e., age, gender, marital status, employment status, whether having children or not, occupation, gaming preference, and the number of times joining game console lotteries) in Supplementary Table 15. Standard errors were clustered by prefectures. The two-sided t-test was used as the statistical test. No adjustments for multiple comparisons were made. Descriptive information on these outcome variables is presented in Supplementary Table 17.

Supplementary Table 7: Comparison of baseline characteristics between respondents and nonrespondents.

Variable	Nonrespondents (N=66,987) Mean/SD	Respondents (N=97,602) Mean/SD	Normalized difference
Age	35.031 [15.489]	38.391 [16.518]	-0.210
Gender (Male)	0.424 [0.494]	0.513 [0.500]	-0.179
Married	0.518 [0.500]	0.572 [0.495]	-0.109
Divorced/separated	0.062 [0.242]	0.064 [0.244]	-0.006
Have child(ren)	0.449 [0.497]	0.502 [0.500]	-0.106
Gaming Preference			
Hardcore gamer	0.215 [0.410]	0.160 [0.367]	0.139
Core gamer	0.234 [0.423]	0.203 [0.402]	0.076
Middle-core gamer	0.214 [0.410]	0.233 [0.423]	-0.045
Casual gamer	0.160 [0.367]	0.176 [0.380]	-0.042
Non-gamer	0.178 [0.382]	0.229 [0.420]	-0.128
Job: industries			
Engineering and construction[1]	0.049 [0.216]	0.054 [0.227]	-0.024
Textile and Cosmetics[2]	0.055 [0.229]	0.055 [0.228]	0.001
Manufacturing	0.088 [0.283]	0.104 [0.305]	-0.053
Trading and Mass media[3]	0.027 [0.161]	0.031 [0.173]	-0.025
Distributors, Retailers	0.048 [0.214]	0.047 [0.212]	0.004
Carriers[4]	0.038 [0.191]	0.041 [0.198]	-0.015
Public works	0.041 [0.199]	0.051 [0.220]	-0.045
IT industries[5]	0.038 [0.192]	0.045 [0.208]	-0.034
Banks and Financial services	0.027 [0.161]	0.028 [0.166]	-0.011
Food and Other Services[6]	0.112 [0.315]	0.109 [0.312]	0.008
Medical care, Welfare	0.080 [0.271]	0.069 [0.253]	0.042
Education	0.034 [0.182]	0.037 [0.188]	-0.013
Others[7]	0.068 [0.252]	0.070 [0.254]	-0.005

Notes. Respondents' characteristics are displayed. Caregivers' characteristics are used where appropriate. The characteristics that were available before sending survey offers are shown.

[1]Civil engineering, Construction, Real estate, Housing and building services; [2]Daily necessaries, Textile and apparel, Cosmetics, Food and Beverages; [3]Trading companies, Publishing, Printing, Mass media; [4]Carriers, Warehousing, Logistics; [5]Software and Information services; [6]Food services, Hairdressing, Cosmetology, Other Services; [7]Other industries and types of business.

Supplementary Table 8: Causal impact of video game engagement on well-being in Japan.

Variables	Number of observations	Model 1		Model 2	
		β coefficient (95% CI)	*P*-value	β coefficient (95% CI)	*P*-value
Outcome variable: Psychological distress (K6)					
Have a Nintendo Switch	1,773	-0.606 (-0.839 to -0.372)	<0.001	-0.602 (-0.824 to -0.38)	<0.001
Played Nintendo Switch this month	1,773	-0.822 (-1.149 to -0.496)	<0.001	-0.814 (-1.099 to -0.53)	<0.001
Have a PS5	6,419	-0.109 (-0.198 to -0.02)	0.017	-0.116 (-0.206 to -0.025)	0.013
Played PS5 this month	6,419	-0.191 (-0.348 to -0.034)	0.018	-0.202 (-0.358 to -0.046)	0.012
Video game play time (hour/day)	3,491	-0.167 (-0.36 to 0.025)	0.088	-0.204 (-0.402 to -0.006)	0.043
Outcome variable: Life satisfaction (SWLS)					
Have a PS5	6,419	0.243 (0.162 to 0.324)	<0.001	0.234 (0.158 to 0.31)	<0.001
Played PS5 this month	6,419	0.425 (0.274 to 0.577)	<0.001	0.408 (0.271 to 0.546)	<0.001
Video game play time (hour/day)	3,491	0.273 (0.067 to 0.478)	0.01	0.265 (0.056 to 0.474)	0.014
Outcome variable: Mild-to-serious psychological distress dummy variable (K6>=5)					
Have a Nintendo Switch	1,773	-0.336 (-0.486 to -0.185)	<0.001	-0.331 (-0.463 to -0.2)	<0.001
Played Nintendo Switch this month	1,773	-0.456 (-0.683 to -0.229)	<0.001	-0.448 (-0.644 to -0.252)	<0.001
Have a PS5	6,419	-0.061 (-0.106 to -0.017)	0.007	-0.072 (-0.111 to -0.033)	<0.001
Played PS5 this month	6,419	-0.107 (-0.186 to -0.029)	0.008	-0.126 (-0.194 to -0.058)	<0.001
Video game play time (hour/day)	3,491	-0.102 (-0.188 to -0.016)	0.02	-0.124 (-0.21 to -0.037)	0.005
Outcome variable: Serious psychological distress dummy variable (K6>=13)					
Have a Nintendo Switch	1,773	-0.179 (-0.297 to -0.062)	0.003	-0.194 (-0.318 to -0.071)	0.002
Played Nintendo Switch this month	1,773	-0.243 (-0.4 to -0.087)	0.002	-0.263 (-0.422 to -0.104)	0.001
Have a PS5	6,419	-0.017 (-0.054 to 0.02)	0.36	-0.025 (-0.062 to 0.012)	0.187
Played PS5 this month	6,419	-0.03 (-0.095 to 0.035)	0.36	-0.043 (-0.108 to 0.021)	0.185
Video game play time (hour/day)	3,491	-0.029 (-0.095 to 0.036)	0.371	-0.037 (-0.108 to 0.033)	0.291

Notes. PS5, PlayStation5; CI, confidence intervals; K6, Kessler psychological distress scale; SWLS, The Satisfaction with Life Scale. The instrumental variable method's estimates are presented. Each estimate used winning Nintendo Switch lottery or PlayStation5 lottery as its instrument, respectively. Exposure variables are presented in each row. Model 1: Adjusted for prefecture dummy variables and round dummy variables only. Model 2: Additionally adjusted for a set of covariates (i.e., age, gender, marital status, employment status, whether having children or not, occupation, gaming preference, and the number of times joining game console lotteries) in Supplementary Table 15. Standard errors were clustered by prefectures. The estimates for K6 and SWLS were standardized by the standard deviations. The two-sided t-test was used as the statistical test. No adjustments for multiple comparisons were made.

Supplementary Table 9: Weak instrument tests.

Variables	Number of observations	Kleibergen-Paap rk Wald F statistic	
		Model 1	Model 2
Outcome variable: Psychological distress (K6)			
Have a Nintendo Switch	1,773	139.8	134.4
Played Nintendo Switch this month	1,773	69.54	76.01
Have a PS5	6,419	1847	1606
Played PS5 this month	6,419	408.8	402.7
Video game play time (hour/day)	3,491	28.85	26.48
Outcome variable: Life satisfaction (SWLS)			
Have a PS5	6,419	1847	1606
Played PS5 this month	6,419	408.8	402.7
Video game play time (hour/day)	3,491	28.85	26.48
Outcome variable: Mild-to-serious psychological distress dummy variable (K6>=5)			
Have a Nintendo Switch	1,773	139.8	134.4
Played Nintendo Switch this month	1,773	69.54	76.01
Have a PS5	6,419	1845	1606
Played PS5 this month	6,419	403	402.7
Video game play time (hour/day)	3,491	28.83	26.48
Outcome variable: Serious psychological distress dummy variable (K6>=13)			
Have a Nintendo Switch	1,773	139.8	134.4
Played Nintendo Switch this month	1,773	69.54	76.01
Have a PS5	6,419	1845	1606
Played PS5 this month	6,419	403	402.7
Video game play time (hour/day)	3,491	28.83	26.48

Notes. PS5, PlayStation5. This table displays weak instrument test statistics corresponding to the models of the instrumental variable method in Supplementary Table 8. Each estimate used winning Nintendo Switch lottery or PlayStation5 lottery as its instrument, respectively. Exposure variables are presented in each row. Model 1: Adjusted for prefecture dummy variables and round dummy variables only. Model 2: Additionally adjusted for a set of covariates (i.e., age, gender, marital status, employment status, whether having children or not, occupation, gaming preference, and the number of times joining game console lotteries) in Supplementary Table 15. The threshold for detecting a weak instrument is <10.

Supplementary Table 10: First-stage regression results of instrumental variable method.

Variables	Number of observations	Model 1		Model 2	
		β coefficient (95% CI)	*P*-value	β coefficient (95% CI)	*P*-value
Outcome variable: Have a Nintendo Switch					
Win Nintendo Switch lottery	1,773	0.323 (0.268 to 0.378)	<0.001	0.305 (0.252 to 0.358)	<0.001
Outcome variable: Played Nintendo Switch this month					
Win Nintendo Switch lottery	1,773	0.238 (0.18 to 0.295)	<0.001	0.226 (0.173 to 0.278)	<0.001
Outcome variable: Have a PS5					
Win PS5 lottery	6,419	0.657 (0.626 to 0.688)	<0.001	0.651 (0.618 to 0.684)	<0.001
Outcome variable: Played PS5 this month					
Win PS5 lottery	6,419	0.375 (0.338 to 0.413)	<0.001	0.373 (0.336 to 0.41)	<0.001
Outcome variable: Video game play time (hour/day)					
Win PS5 lottery	3,491	0.569 (0.356 to 0.782)	<0.001	0.538 (0.328 to 0.749)	<0.001

Notes. PS5, PlayStation5; CI, confidence intervals. This table displays first-stage regression results corresponding to the models in Supplementary Table 8. Model 1: Adjusted for prefecture dummy variables and round dummy variables only. Model 2: Additionally adjusted for a set of covariates, including gaming preferences. Standard errors were clustered by prefectures. The two-sided t-test was used as the statistical test. No adjustments for multiple comparisons were made.

Supplementary Table 11: Major theories, mechanisms, and hypotheses in literature.

Effect	Theory / mechanism / hypothesis	Brief overview	Related empirical papers
Positive	Self-determination theory	Any activity whose affordances align with the motivations of people will contribute to their well-being. If an activity also satisfies basic psychological needs for competence, relatedness and autonomy, people will find the activity more motivating, enjoyable and immersive, leading to higher well-being.	Ryan, Rigby, and Przybylski[23]
Positive	Mood management theory	People use the media to induce desired moods.	Whitaker and Bushman[24]
Positive	Game genre hypothesis: exergames and relaxing games	Exergames (or fitness games) can enhance one's mental well-being through physical exercise. Relaxation games promote mental well-being by creating a positive mood, as used in psychological therapy.	Primack et al.[25]
Positive	Combating loneliness through communication	Online video games present an opportunity for players to foster social connections by engaging in playing with friends or with people online, thereby alleviating feelings of solitude and promoting positive mental health. The use of gaming as a means of social compensation may alleviate emotional distress experienced during the pandemic-induced self-isolation.	Pallavicini, Pepe, and Mantovani[26]
Negative	Reduction of sleep time	Insufficient sleep or worse sleep quality caused by video gaming is harmful to mental well-being.	Peracchia and Curcio[27]
Negative	Increase of sedentary behavior / reduction of exercise time	Prolonged sedentary behavior can negatively impact mental well-being.	Suchert, Hanewinkel, and Isensee[28]
Negative	Reduction of offline social interactions	Video gaming (or online communications) could reduce face-to-face social interactions and potentially harm mental well-being.	Kowert et al.[29]

Supplementary Table 12: Number of observations for each round.

Round	# of people that were sent survey offers	# of people that answered survey	# of people who joined lotteries	# of people who won lotteries	Switch lotteries or PS5 lotteries
1	34,615	18,912	1,773	926	Switch
2	27,186	18,189	1,481	254	PS5
3	29,462	18,479	1,447	332	PS5
4	47,183	26,996	2,127	499	PS5
5	26,143	15,026	1,364	312	PS5

Notes. Switch, Nintendo Switch; PS5, PlayStation5. The response rate was 59.3 percent (97,602/164,589). In total, 8,192 people joined the game console lotteries.

Supplementary Table 13: Exposures: variables capturing game engagement.

Variables	Source and notes
Nintendo Switch Ownership	Respondents were asked whether their household owned a Nintendo Switch. A dichotomous variable is used in statistical analysis.
PS5 Ownership	Respondents were asked whether their household owned a PS5. A dichotomous variable is used in statistical analysis.
Nintendo Switch Usage	Respondents were asked whether they had played Nintendo Switch over the last 30 days. A dichotomous variable is used in statistical analysis.
PS5 Usage	Respondents were asked whether they had played PS5 over the last 30 days. A dichotomous variable is used in statistical analysis.
Time spent playing video games	Time of video game play, including any video games on TV/computer, in hours per day. Respondents were asked how much time they spent playing video games on the weekdays (and, in another question, the weekends) over the past 30 days.
Time spent playing smartphone games	Time of playing smartphone games, in hours per day. Respondents were asked how much time they spent playing smartphone games on the weekdays (and, in another question, the weekends) over the past 30 days.

Notes. PS5, PlayStation5.

Supplementary Table 14: Outcomes and exposures collected in each round.

Outcomes and exposures	Round				
	1	2	3	4	5
	Switch	PS5	PS5	PS5	PS5
K6	✓	✓	✓	✓	✓
SWLS		✓	✓	✓	✓
Video game play time				✓	✓
Have Nintendo Switch	✓	✓	✓	✓	✓
Play Nintendo Switch this month	✓	✓	✓	✓	✓
Have PS5	✓	✓	✓	✓	✓
Play PS5 this month	✓	✓	✓	✓	✓

Notes. Switch, Nintendo Switch; PS5, PlayStation5; K6, Kessler psychological distress scale; SWLS, The Satisfaction with Life Scale. Round one survey collected Nintendo Switch lotteries' information. From round two, we collected PS5 lotteries' information.

Supplementary Table 15: Description of covariates.

Variables	Source and notes
Age	Age of respondents in years.
Gender	Male or female. In statistical analysis, the male gender was encoded as 1, while the female gender was encoded as 0.
Marital status	Married, unmarried, or divorced/separated. In statistical analysis, dichotomous variables were used, and each category was encoded as 1 and otherwise encoded as 0.
Having children	Yes or No. In statistical analysis, having children was encoded as 1 and otherwise 0.
Employment status	Full-time employee, part-time employee, student, stay-at-home wife/husband, unemployed, or self-employed/others. In statistical analysis, dichotomous variables were used, and each category was encoded as 1 and otherwise encoded as 0.
Video gaming preference	Hardcore gamers, Core gamers, Mid-core gamers, Casual gamers, or Non-gamers. The level of affection for the game declines in the order listed. In statistical analysis, each category was encoded as 1 and otherwise encoded as 0. The preference was assessed with a clustering algorithm by the research company GRI based on frequencies of game play and game software purchase, and categorized into five groups. The preference was measured in November 2019, where available. If not available, the one assessed in November 2020 or November 2021 was employed. This is to avoid bad control issues.
Number of times joining game console lotteries	The number of times respondent households joined Nintendo Switch lotteries and PS5 lotteries were measured, respectively. To obtain estimates more robust to outliers, we winsorized (a statistical technique meaning replacement of outliers by less extreme values) the number of times joining lotteries at the top 1 percent (see, for example, Yale and Forsythe[30]).
Occupation	Occupation of respondents/caregivers. We categorized into 14 categories: 'Civil engineering, Construction, Real estate, Housing and building services', 'Daily necessaries, Textile and apparel, Cosmetics, Food and Beverages', 'Manufacturing', 'Trading companies, Publishing, Printing, Mass media', 'Distributors, Retailers', 'Carriers, Warehousing, Logistics', 'Public works', 'Software and Information services', 'Banks and Financial services', 'Food services, Hairdressing, Cosmetology, Other Services', 'Medical care, Welfare', 'Education', 'Other industries and types of business', and 'Not applicable/No answer.' Initially, the survey included 37 categories, which the authors reclassified. In statistical analysis, dichotomous variables were used, and every 14 types were encoded as 1 and otherwise encoded as 0.
Prefecture	Prefecture of residence. In statistical analysis, dichotomous variables were used, and every 47 prefectures were encoded as 1 and otherwise encoded as 0.

Notes. GRI, gameage R&I.

Supplementary Table 16: List of covariates used as features in causal forest algorithm.

Variable description	Type	Variable description	Type
Age	Continuous	Number of times joining game console lotteries	Continuous
Gender (Male)	Binary	**Number of lottery entries per household members**	
Have children (yes)	Binary	Respondent him/herself participated.	Continuous
**Marital status**		Brothers/sisters participated.	Continuous
Married	Binary	Children participated.	Continuous
Divorced/separated	Binary	Parents/grandparents participated.	Continuous
Not married	Binary	Other siblings participated.	Continuous
**Occupation**		**Number of lottery entries per stores**	
Student	Binary	Online lottery at Sony Store.	Continuous
Stay-at-home wife/husband	Binary	Online lottery at Nintendo Store.	Continuous
Full-time employee	Binary	Online lottery at GEO.	Continuous
Part-time employee	Binary	Online lottery at Bic Camera.	Continuous
Self employed/others	Binary	Online lottery at Yamada Denki.	Continuous
Unemployed/not a student	Binary	Online lottery at Yodobashi Camera.	Continuous
**Gaming preference**		Online lottery at Joshin.	Continuous
Hardcore gamer	Binary	Online lottery at Edion.	Continuous
Core gamer	Binary	Online lottery at Sofmap.	Continuous
Middle-core gamer	Binary	Online lottery at Nojima.	Continuous
Casual gamer	Binary	Online lottery at Aeon.	Continuous
Non-gamer	Binary	Online lottery at Don Quijote.	Continuous
**Job: Industries**		Online lottery at Otakarasouko.	Continuous
Engineering and construction	Binary	Online lottery at Furuhon-ichiba.	Continuous
Textile and Cosmetics	Binary	Online lottery at other stores.	Continuous
Manufacturing	Binary	Over-the-counter lottery at TSUTAYA.	Continuous
Trading and Mass media	Binary	Over-the-counter lottery at Kojima.	Continuous
Distributors, Retailers	Binary	Over-the-counter lottery at Edion.	Continuous
Carriers	Binary	Over-the-counter lottery at Sofmap.	Continuous
Public works	Binary	Over-the-counter lottery at Yodobashi Camera.	Continuous
IT industries	Binary	Over-the-counter lottery at other stores.	Continuous
Banks and Financial services	Binary	Lottery at any other stores.	Continuous
Food and Other Services	Binary		
Medical care, Welfare	Binary		
Education	Binary		
Others	Binary		
Not applicable	Binary		
Indicator of being answered by caregivers on behalf of children.	Binary		
47 Prefecture indicators for each	Binary		
5 round indicators for each	Binary		

Notes. As for the marital status (unmarried, married, divorced/separated) and the indicator of having children (yes, no), we employed two types of variables. One set of variables pertains to the attributes of either the respondents or caregivers, while the other set of variables relates to the respondents' characteristics.

Supplementary Table 17: Outcomes by exposure.

	Psychological distress (K6)				Life satisfaction (SWLS)	
	N	Mean (SD)	Mild-to-serious psychological distress (K6>=5) (%)	Serious psychological distress (K6>=13) (%)	N	Mean (SD)
**Win a Nintendo Switch lottery**						
Yes	926	7.6 (7.2)	55.1	26.6		
No	847	9.0 (7.4)	65.3	31.9		
Total	1,773	8.2 (7.3)	60.0	29.1		
**Win a PS5 lottery**						
Yes	1,397	6.8 (6.9)	51.3	21.3	1,397	17.8 (6.9)
No	5,022	7.3 (6.9)	55.8	23.3	5,022	16.6 (6.6)
Total	6,419	7.2 (6.9)	54.8	22.8	6,419	16.9 (6.6)
**Have a Nintendo Switch**						
Yes	28,620	5.4 (6.1)	43.2	14.0	23,860	17.7 (6.4)
No	68,982	5.6 (6.2)	44.7	14.3	54,830	16.8 (6.6)
**Played Nintendo Switch this month**						
Yes	17,890	5.6 (6.3)	44.6	14.9	14,860	17.8 (6.5)
No	79,712	5.5 (6.2)	44.2	14.1	63,830	16.9 (6.6)
**Have a PS5**						
Yes	2,229	6.5 (7.0)	48.7	20.0	1,960	18.1 (6.8)
No	95,373	5.5 (6.2)	44.1	14.1	76,730	17.1 (6.5)
**Played PS5 this month**						
Yes	1,202	6.2 (6.8)	46.4	18.6	1,062	18.5 (6.7)
No	96,400	5.5 (6.2)	44.2	14.1	77,628	17.1 (6.5)
Total	97,602	5.5 (6.2)	44.2	14.2	78,690	17.1 (6.6)
**Video game play time**						
<1 hour/day	28,842	5.0 (5.9)	40.9	11.9	28,842	17.1 (6.6)
1-3 hours/day	10,871	5.5 (6.1)	44.7	13.8	10,871	17.3 (6.5)
More than 3 hours/day	2,309	6.3 (6.6)	48.5	19.2	2,309	16.4 (7.0)
**Smartphone game play time**						
<1 hour/day	23,364	4.9 (5.9)	39.7	11.7	23,364	17.1 (6.6)
1-3 hours/day	16,027	5.4 (6.0)	44.4	13.2	16,027	17.2 (6.4)
More than 3 hours/day	2,631	6.8 (6.7)	52.8	20.4	2,631	16.4 (6.9)
Total	42,022	5.2 (6.0)	42.3	12.8	42,022	17.1 (6.6)

Notes. PS5, PlayStation5.

Supplementary Figures.

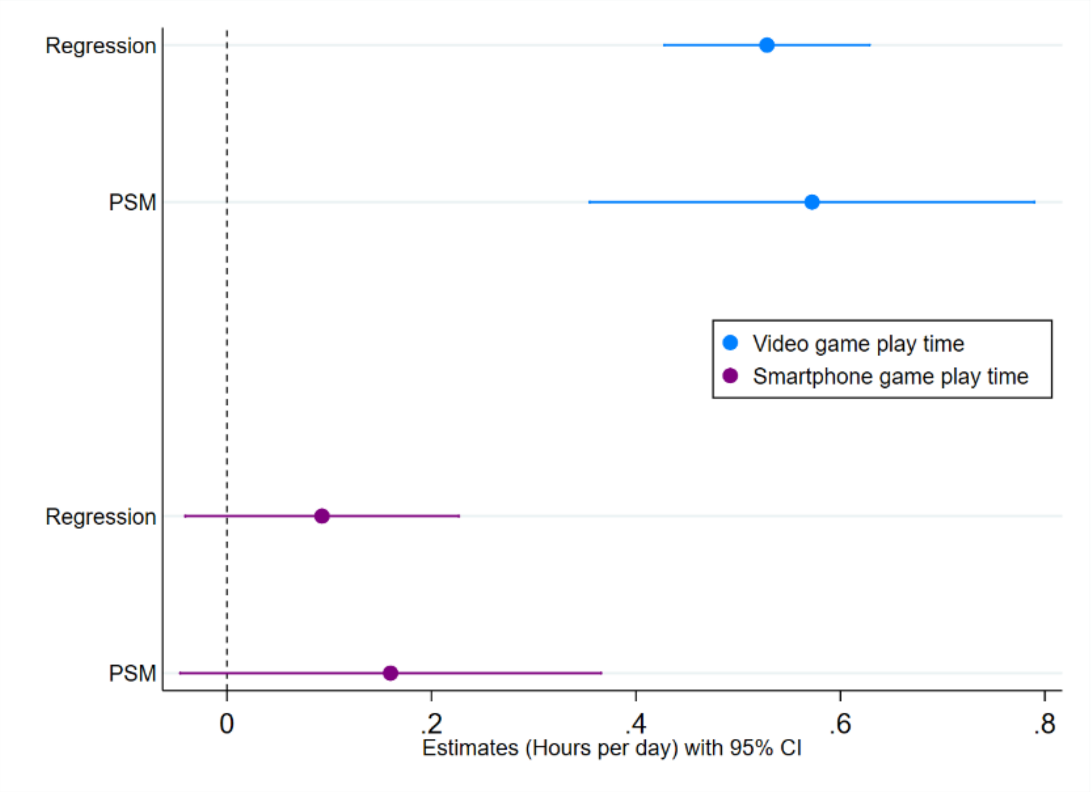

Supplementary Figure 1: Causal effect of winning game console lotteries on gaming time (N=3,491).

Notes. CI, confidence interval; PSM, Propensity Score Matching. The analysis sample was limited to those who joined game console lotteries and responded in rounds 4-5. The point estimates (mean values) and the 95 percent confidence intervals are shown. Abadie-Imbens robust standard errors were used for PSM. Equation (1) was used for the regression estimates (Supplementary Methods). Standard errors were clustered by prefectures.

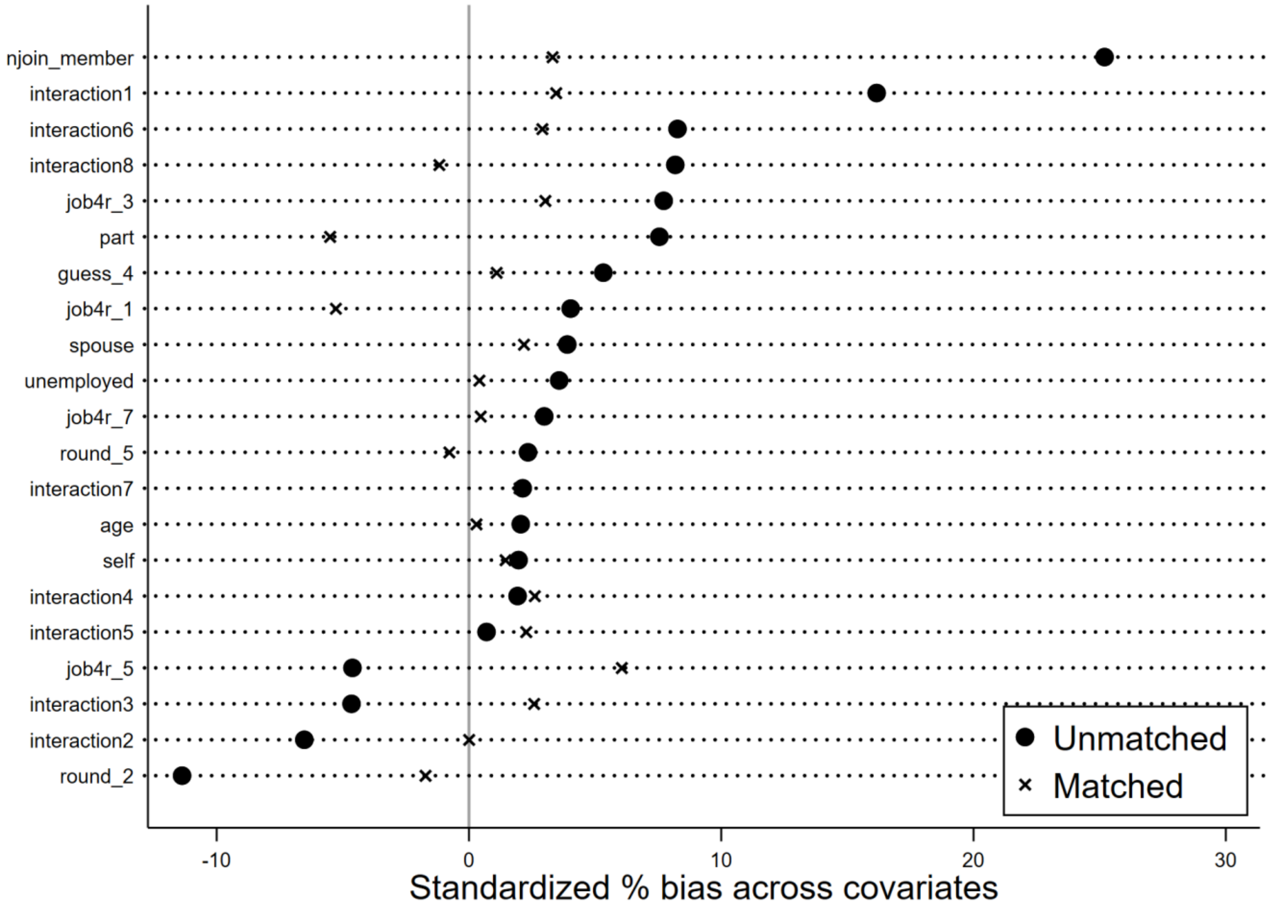

Supplementary Figure 2: Balance check before and after propensity score matching for estimations regarding PlayStation5.

Notes. njoin_member: The number of times a household joined PlayStation5 lotteries.
age: Age in number; guess_4: A dummy variable indicating a casual gamer.
spouse: A dummy variable indicating a spouse.
part: A dummy variable indicating a part-time employee; self: A dummy variable indicating self-employed.
unemployed: A dummy variable indicating unemployed.
job4r_1: A dummy variable indicating that he/she works for the engineering and construction industry.
job4r_3: A dummy variable indicating that he/she works for the manufacturing industry.
job4r_5: A dummy variable indicating that he/she works for distributors and retailers.
job4r_7: A dummy variable indicating that he/she works for public works.
round_2: A dummy variable indicating that the observation is collected in the survey round 2.
round_5: A dummy variable indicating that the observation is collected in the survey round 5.
interaction1: njoin member * njoin member
interaction2: job4r_3 * round 2
interaction3: round_5 * self
interaction4: job4r_7 * njoin member
interaction5: job4r_5 * round 2
interaction6: spouse * guess 4
interaction7: age * spouse
interaction8: job4r_3 * part

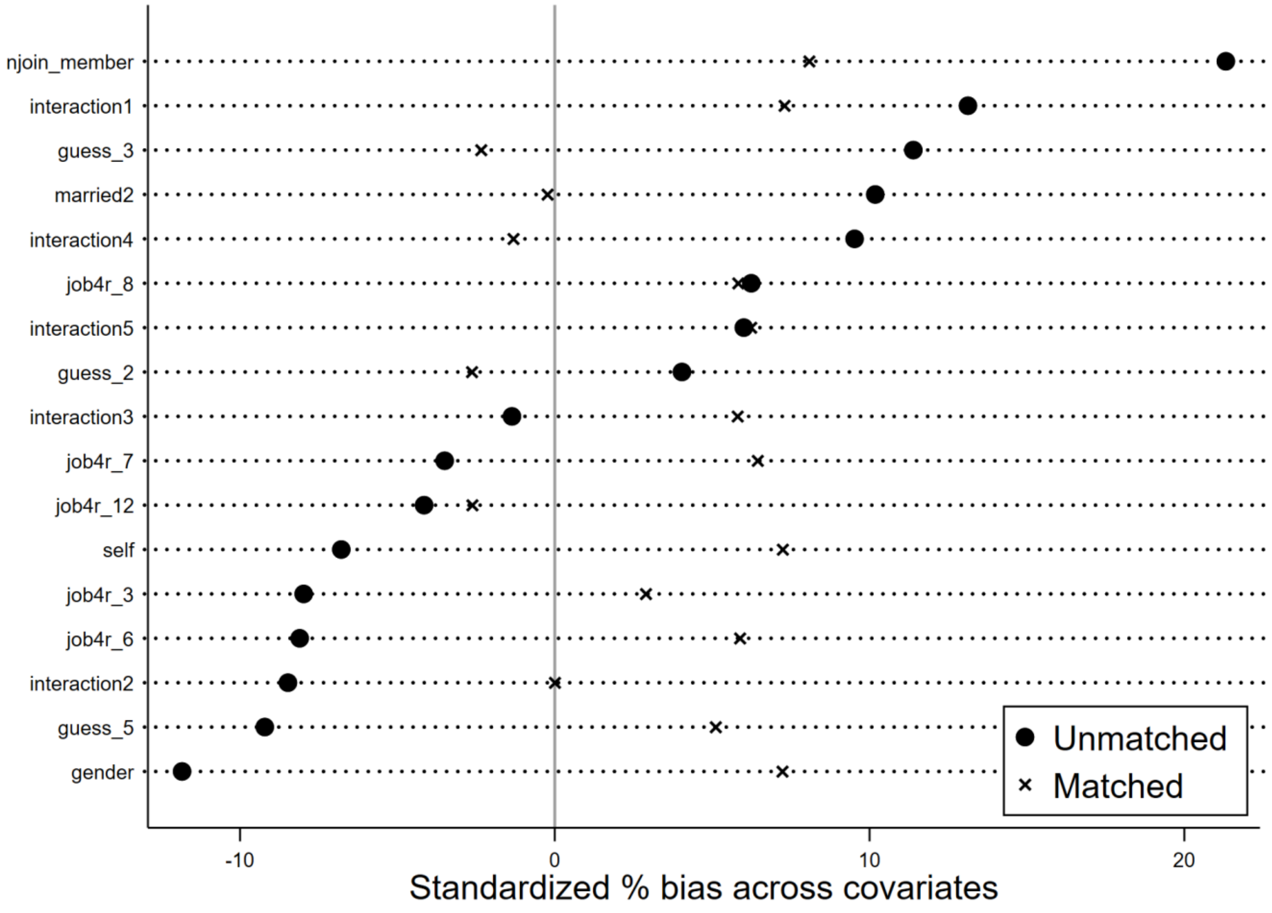

Supplementary Figure 3: Balance check before and after propensity score matching for estimations regarding Nintendo Switch.

Notes. njoin_member: The number of times a household joined Nintendo Switch lotteries.
gender: A dummy variable indicating a male.
guess_2: A dummy variable indicating a core gamer.
guess_3: A dummy variable indicating a middle-core gamer.
guess_5: A dummy variable indicating a non-gamer.
married2: A dummy variable indicating that either respondents or caregivers are married.
self: A dummy variable indicating self-employed.
job4r_3: A dummy variable indicating that he/she works for the manufacturing industry.
job4r_6: A dummy variable indicating that he/she works for the carriers, warehousing, and logistics industry.
job4r_7: A dummy variable indicating that he/she works for public works.
job4r_8: A dummy variable indicating that he/she works for the IT industry.
job4r_12: A dummy variable indicating that he/she works for the education industry.
interaction1: njoin_member * njoin_member
interaction2: job4r_6 * guess_2
interaction3: job4r_6 * married2
interaction4: guess_2 * married2
interaction5: self * njoin_member

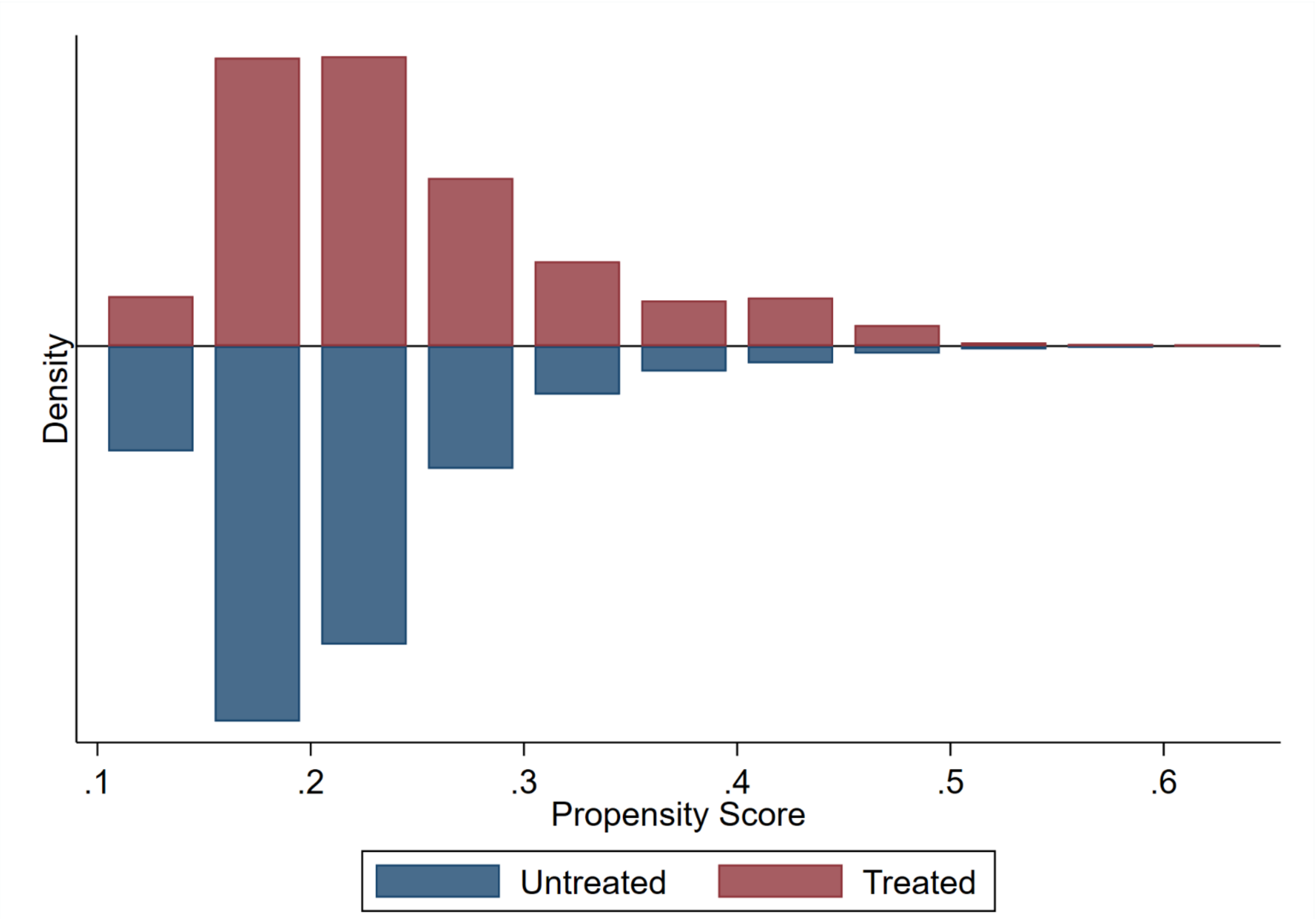

Supplementary Figure 4: Common support check for propensity score matching estimations regarding PlayStation5.

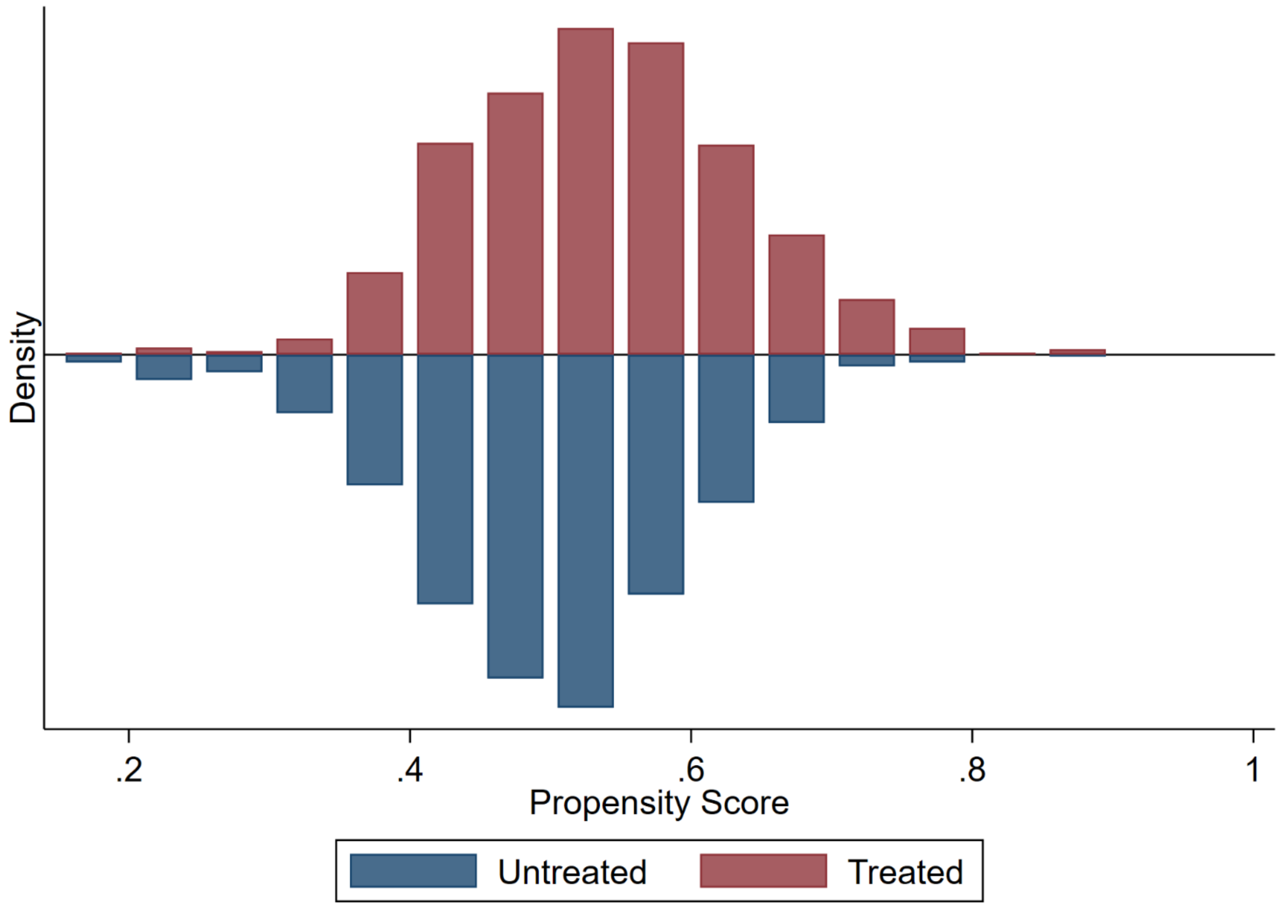

Supplementary Figure 5: Common support check for propensity score matching estimations regarding Nintendo Switch.

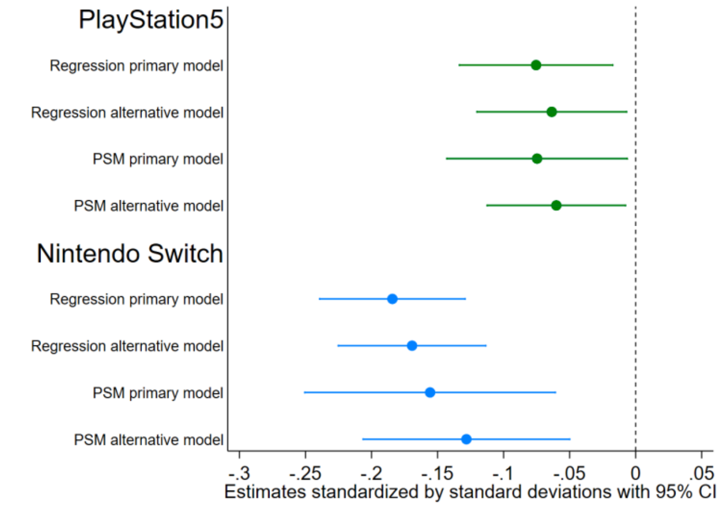

(a) Psychological distress (K6)

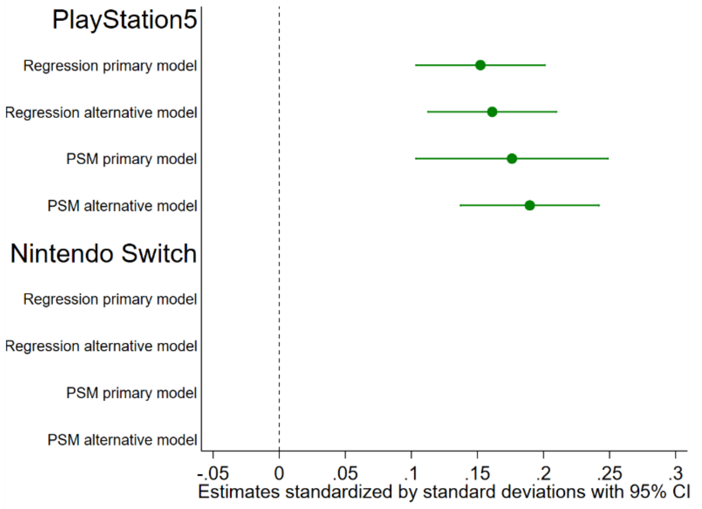

(b) Life satisfaction (SWLS)

Supplementary Figure 6: Intention-to-Treat estimates using Imputed-Console-Purchase-Motivation instead of the number of times of lottery participation (N=8,192).

Notes: CI, confidence intervals; PSM, Propensity Score Matching. The analysis sample is limited to those who joined game console lotteries. The point estimates (mean values) and the 95 percent confidence intervals are shown. Regression standard errors are clustered by prefectures. Abadie-Imbens robust standard errors are used for PSM. As for the primary model, Equation (1) is used for the regression estimates (Supplementary Methods). The number of times of lottery participation was replaced by the imputed-console-purchase-motivation for the alternative model. A lower K6 means having less psychological distress, while a higher SWLS means greater life satisfaction. The estimates are standardized by the standard deviations. The random forest model, used for the imputation, was constructed using R's Random Forest package, configured with 500 trees, terminal node sizes of at least five, and considering $\frac{K}{3}$ covariates at each split, where K represents the total number of predictors; a 10-fold cross-validation segmented the dataset into 10 subsets.

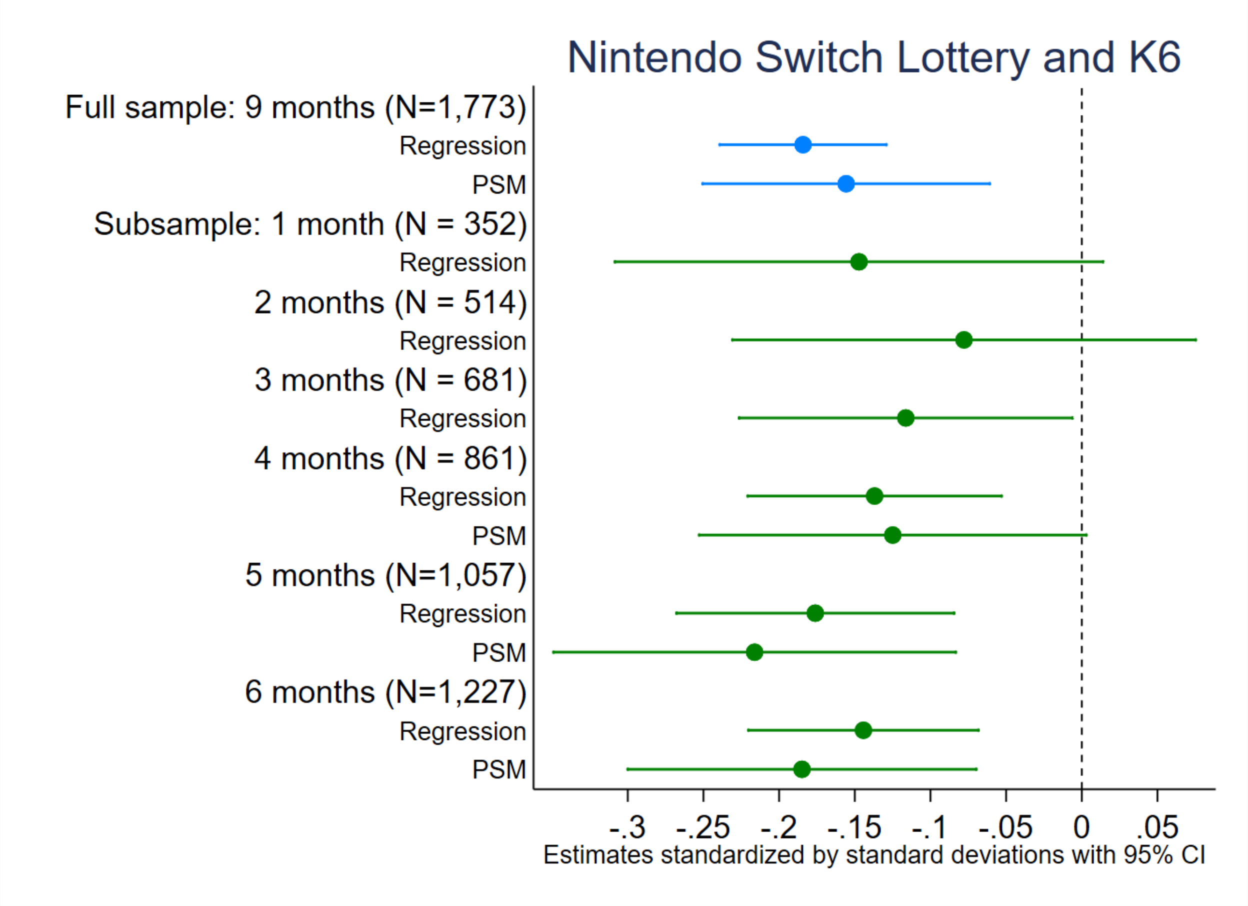

Supplementary Figure 7: Intention-to-Treat estimates using subsample of short period data.
Notes: K6, Kessler psychological distress scale; CI, confidence intervals; PSM, Propensity Score Matching. The analysis sample is limited to those who joined Nintendo Switch lotteries. The point estimates (mean values) and the 95 percent confidence intervals are shown. Regression standard errors are clustered by prefectures. Abadie-Imbens robust standard errors are used for PSM. As for the primary model, Equation (1) is used for the regression estimates (Supplementary Methods), while the number of times of lottery participation is restricted to the period of interest. The outcome variable is K6; a lower K6 means having less psychological distress. The estimates are standardized by the standard deviations.

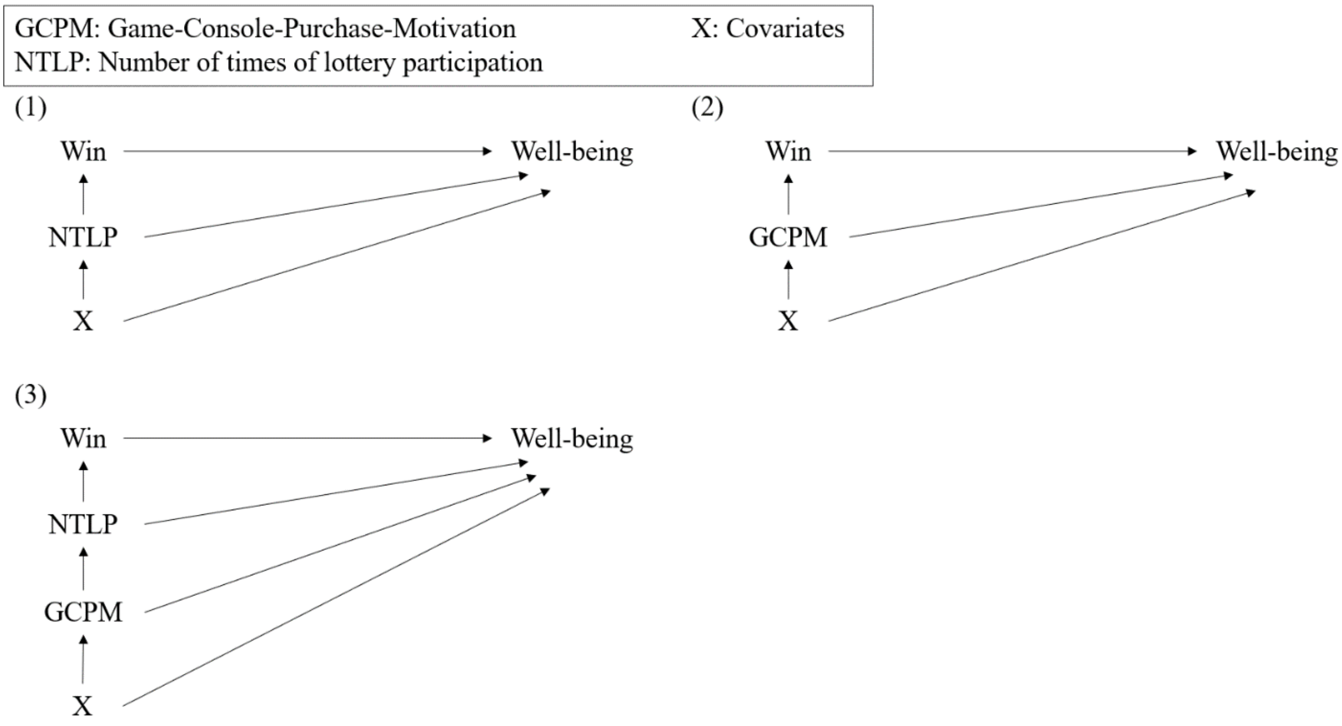

Supplementary Figure 8: Causal diagrams representing Intention-to-Treat analysis.

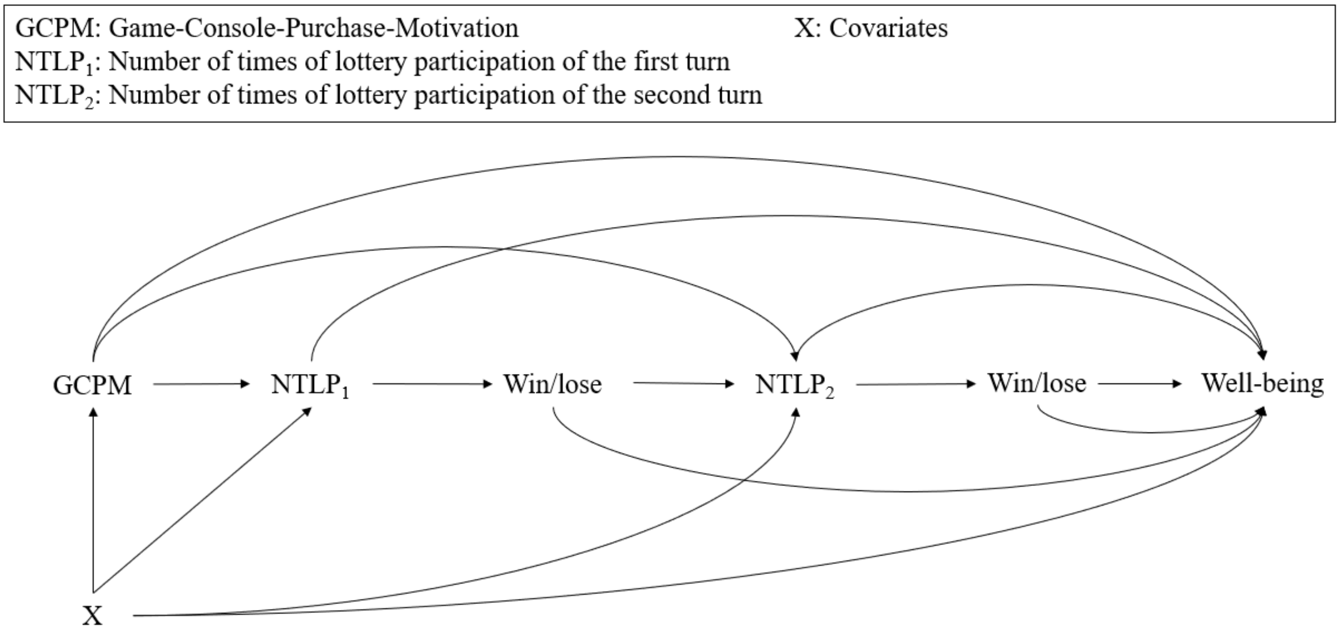

Supplementary Figure 9: Causal diagram with two 'turns' representing Intention-to-Treat analysis.

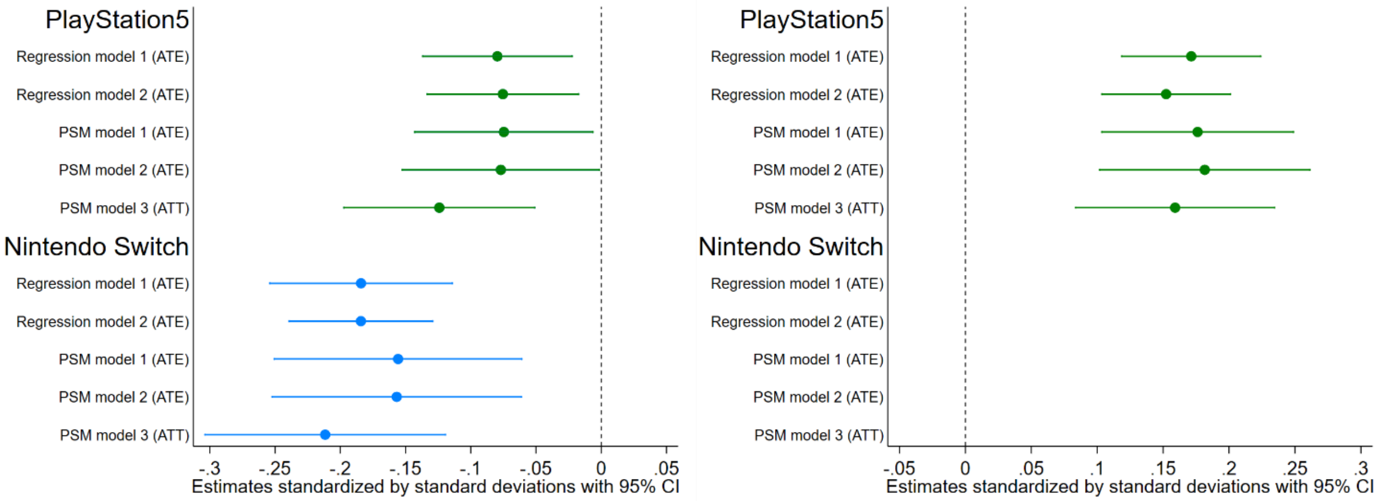

(a) Psychological distress (K6) (b) Life satisfaction (SWLS)

Supplementary Figure 10: Sensitivity checks for estimating impact of winning game console lottery on well-being (N=8,192).

Notes. CI, confidence intervals; PSM, Propensity Score Matching; ATE, Average Treatment Effect; ATT, Average Treatment Effect of the Treated. In this figure, we use the terms ATE and ATT to avoid confusion; ATE is the same as the Intention-to-Treat effect in the main text, while ATT could be formally expressed as the Intention-to-Treat effect on the assigned treatment group. The analysis sample was limited to those who joined game console lotteries. The point estimates (mean values) and the 95 percent confidence intervals are shown. Abadie-Imbens robust standard errors were used for PSM. Regression standard errors were clustered by prefectures. A lower K6 means having less psychological distress, while a higher SWLS means greater life satisfaction. The estimates were standardized by the standard deviations. Regression model 1: No adjustment. Regression model 2 (the principal model used in the main text): Adjusted for a set of covariates (i.e., age, gender, marital status, employment status, whether having children or not, occupation, gaming preference, and the number of times joining game console lotteries) in Supplementary Table 15, prefecture dummy variables, and round dummy variables. PSM model 1 (the principal model used in the main text): Two-to-one nearest neighbor matching with replacement, estimating ATE. PSM model 2: One-to-one nearest neighbor matching with replacement, estimating ATE. PSM model 3: One-to-one nearest neighbor matching without replacement, estimating ATT.

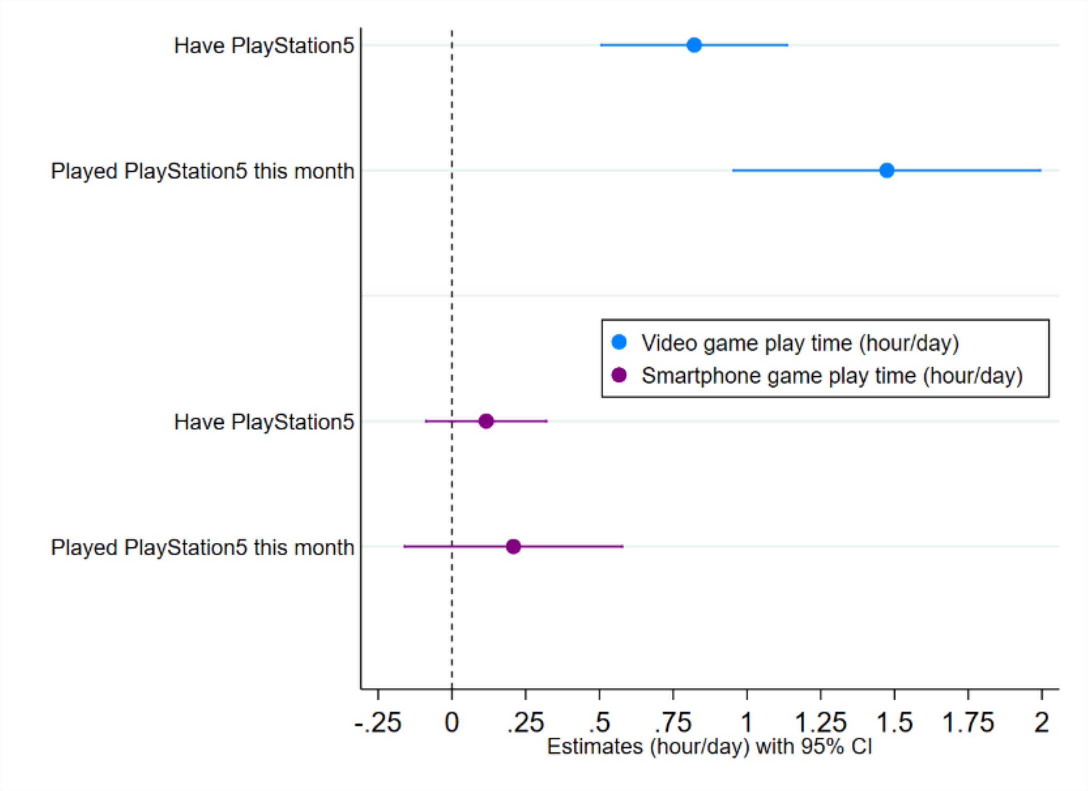

Supplementary Figure 11: Causal effect of ownership of PS5 on gaming time estimated by instrumental variable method (N=3,491).

Notes. CI, confidence intervals; PS5, PlayStation5. The analysis sample was limited to those who joined game console lotteries and responded in rounds 4-5. The point estimates (mean values) and the 95 percent confidence intervals are shown. Standard errors were clustered by prefectures.

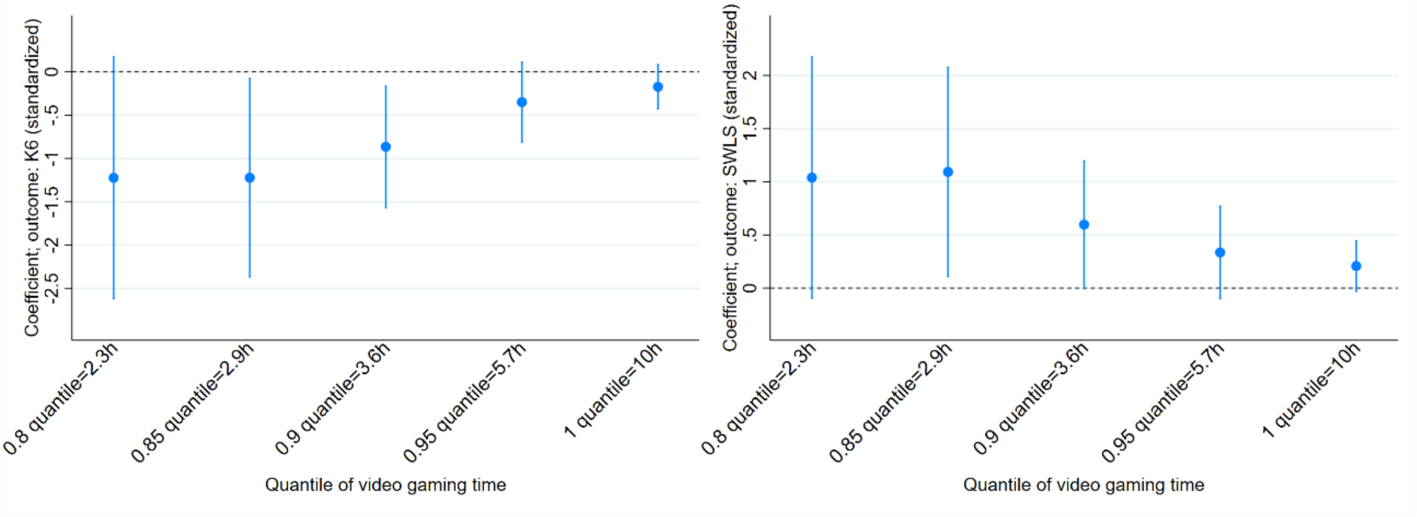

(a) Prolonged video gaming effect on K6

(b) Prolonged video gaming effect on SWLS

Supplementary Figure 12: Subgroup analysis of treatment effect using instrumental variable method.

Notes. K6, Kessler psychological distress scale; SWLS, The Satisfaction with Life Scale. Treatment effects for subgroups were illustrated, taking β coefficients as the y-axis and the quantile of exposure variables as the x-axis. A lower K6 means having less psychological distress, while a higher SWLS means greater life satisfaction. Subgroup instrumental variable regression's bandwidths are 60 percent. Each figure shows estimates based on five subgroups: (i) 0.2 to 0.8 quantile (0.3 to 2.3 hours; n=1,762), (ii) 0.25 to 0.85 quantile (0.3 to 2.9 hours; n=1,892), (iii) 0.3 to 0.9 quantile (0.3 to 3.6 hours; n=2,068), (iv) 0.35 to 0.95 quantile (0.6 to 5.7 hours; n=2,091), and (v) 0.4 to 1.00 quantile (0.9 to 10 hours; n=2,016). The exposure variable is video gaming time. All five group estimates have no weak instrument problem (Kleibergen-Paap F statistics: (i)-(v) 12.74, 16.58, 33.61, 22.02, 15.45. The threshold for detecting a weak instrument is <10.). Regression standard errors were clustered by prefectures. The point estimates (mean values) and the 95 percent confidence intervals are shown.

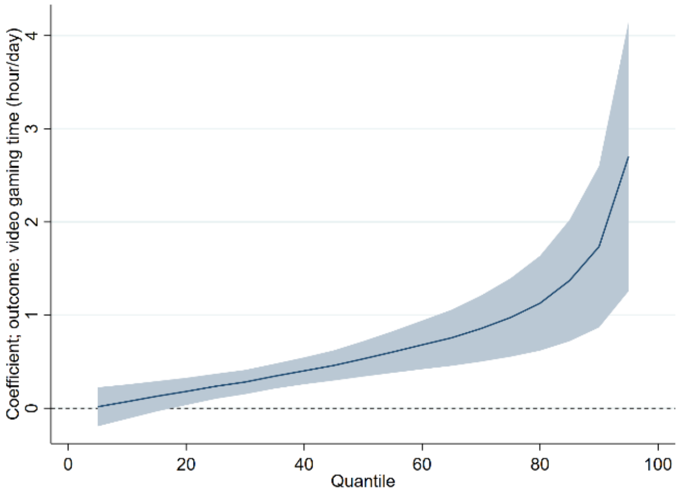

(a) PlayStation5 ownership effect on gaming time

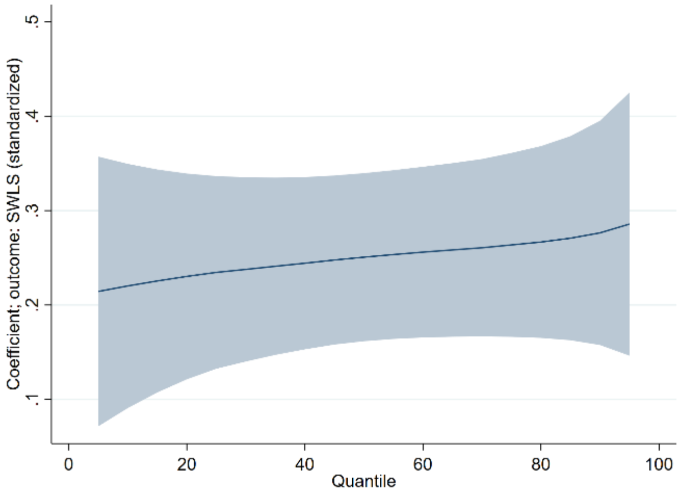

(b) PlayStation5 ownership effect on SWLS

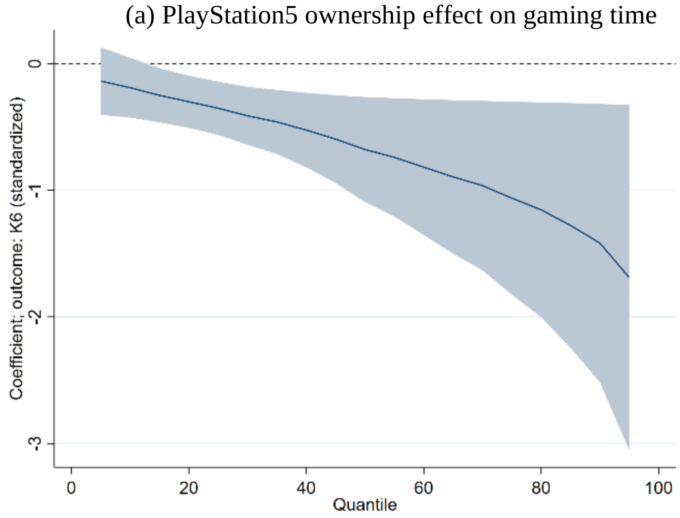

(c) Nintendo Switch ownership effect on K6

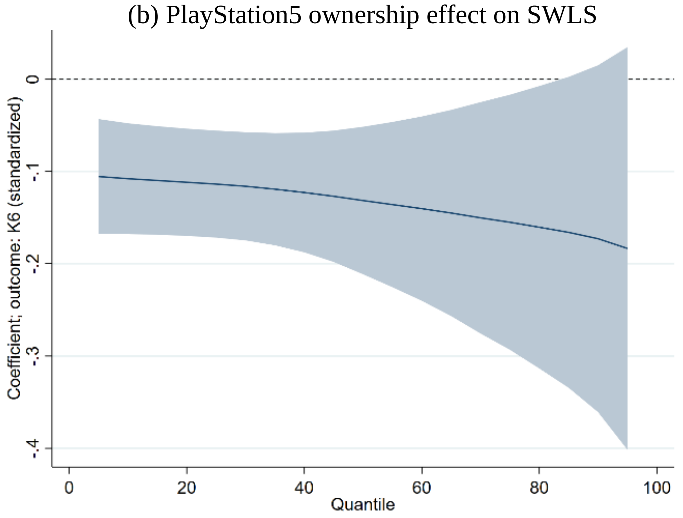

(d) PlayStation5 ownership effect on K6

Supplementary Figure 13: Quantile treatment effect analysis.

Notes. QTE, Quantile treatment effect; K6, Kessler psychological distress scale; SWLS, The Satisfaction with Life Scale. QTEs are illustrated, taking QTE (β coefficient) as the y-axis and the quantile of outcome variables as the x-axis. The instrumental variable model included a set of covariates (for example, round dummy variable, the number of times joining lotteries, and gaming preference dummy variables), yet prefecture dummy variables were excluded due to convergence problems when calculating. Standard errors were estimated by bootstrap. The point estimates (mean values) and the 95 percent confidence intervals are shown. The estimates are standardized by the standard deviations.

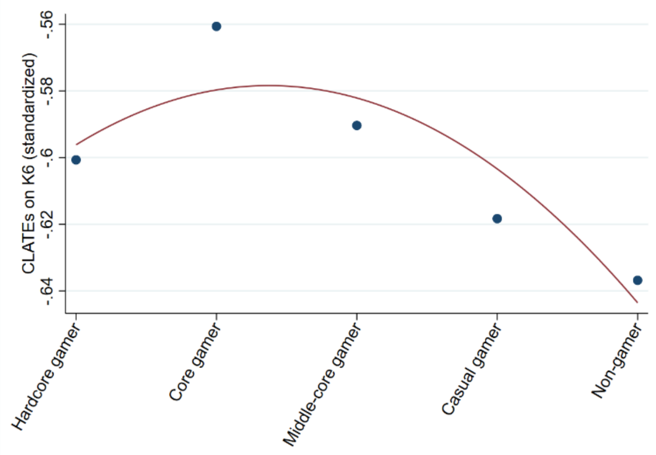

(a) Nintendo Switch

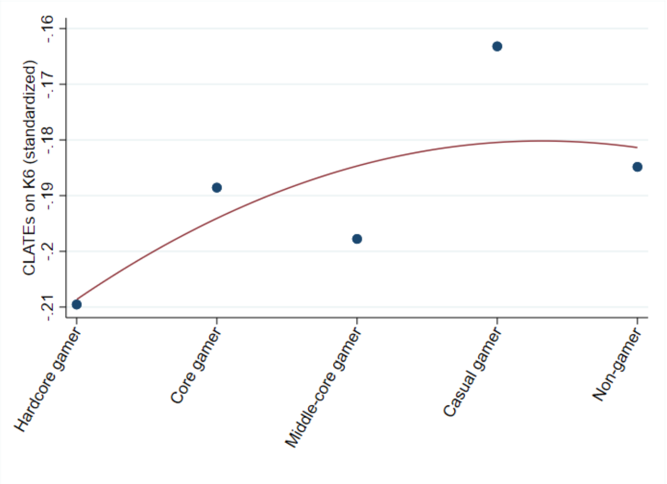

(b) PlayStation5

Supplementary Figure 14: Effect modification of video game ownership by gaming preference (N=8,192).

Notes. CLATE, Conditional local average treatment effect; K6, Kessler psychological distress scale. The instrumental variable causal forest was used to estimate conditional local average treatment effects (conditional LATEs) on K6. Means for each video gaming preference category are plotted. The CLATEs were standardized by the standard deviations.

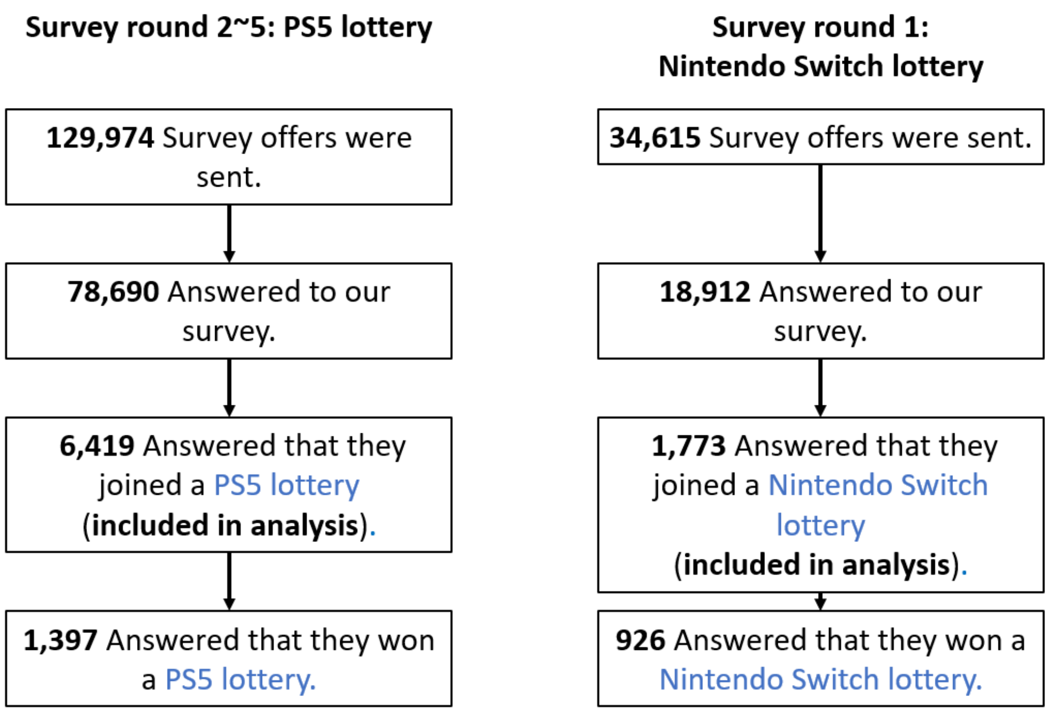

Supplementary Figure 15: Survey participants and analysis sample.

Notes. PS5, PlayStation5. The analysis sample for our causal inference comprised 8,192 (=6,419 + 1,773) individuals.

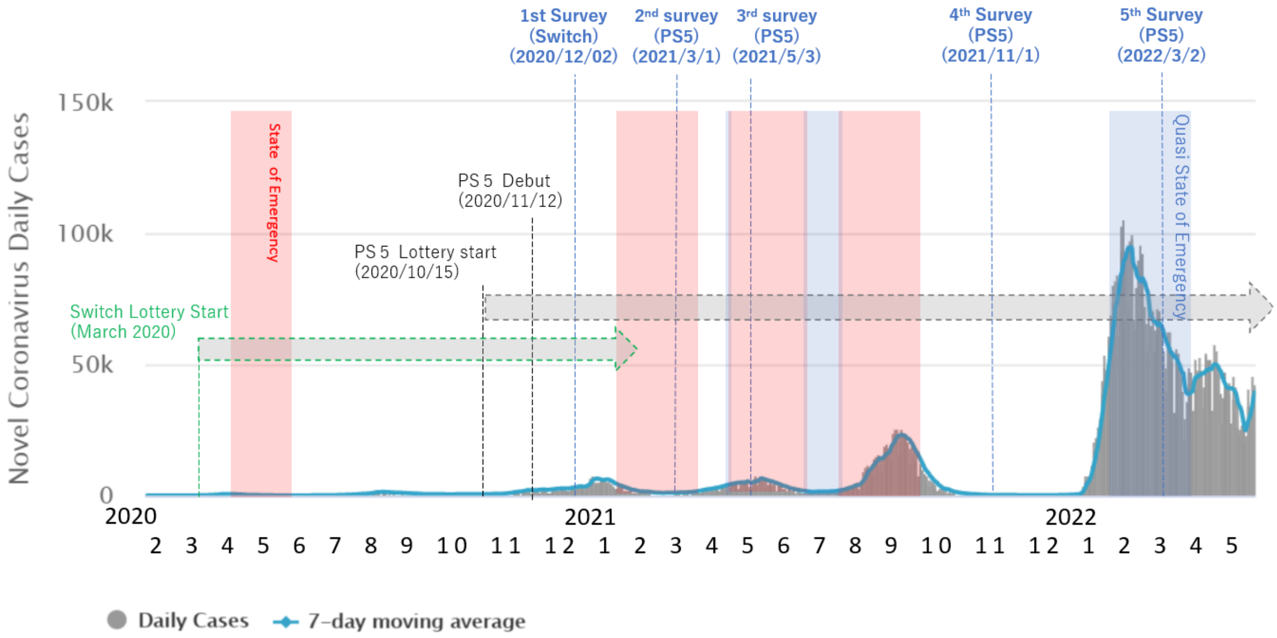

Supplementary Figure 16: Survey schedule and video game consoles' lotteries.

Notes. Red shadow indicates the period of the State of Emergency in Japan due to the COVID-19 pandemic. Blue shadow indicates the Quasi State of Emergency. Source: https://www.worldometers.info/coronavirus/country/japan/

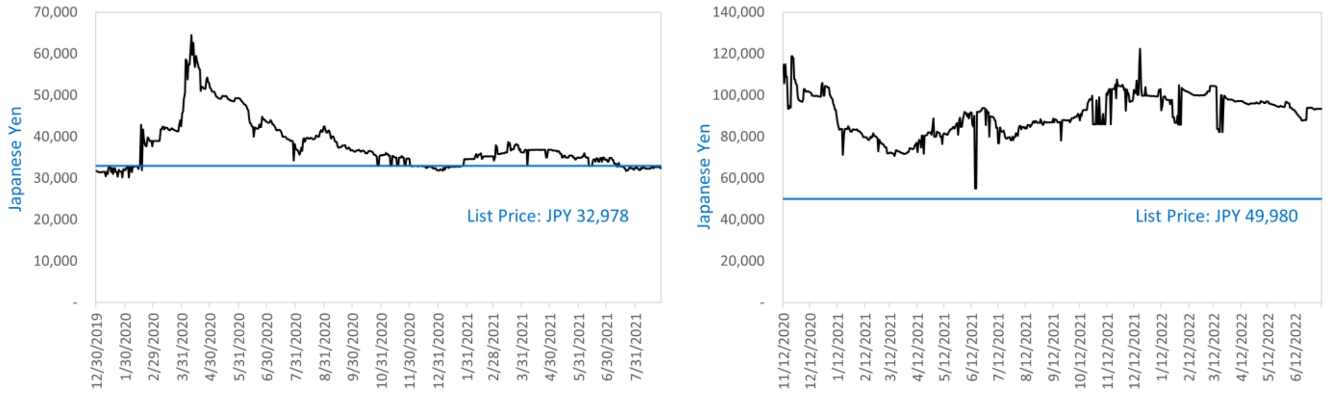

(a) Nintendo Switch (b) PS5

Supplementary Figure 17: Price history of video game consoles (Japanese Yen).

Notes. PS5, PlayStation5. The lowest prices are displayed. Source: https://kakaku.com/

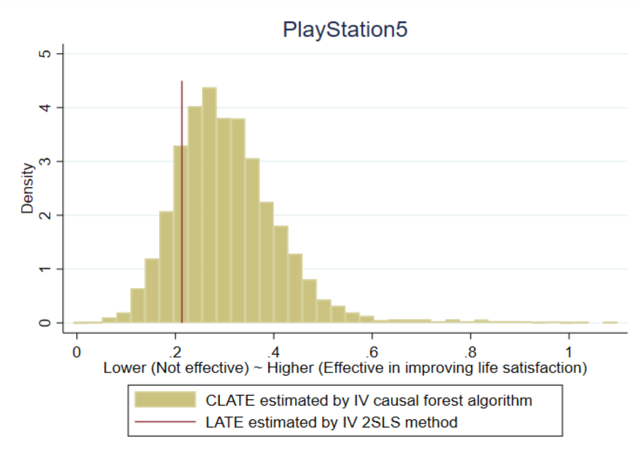

(a) CLATE of ownership of PS5 on life satisfaction

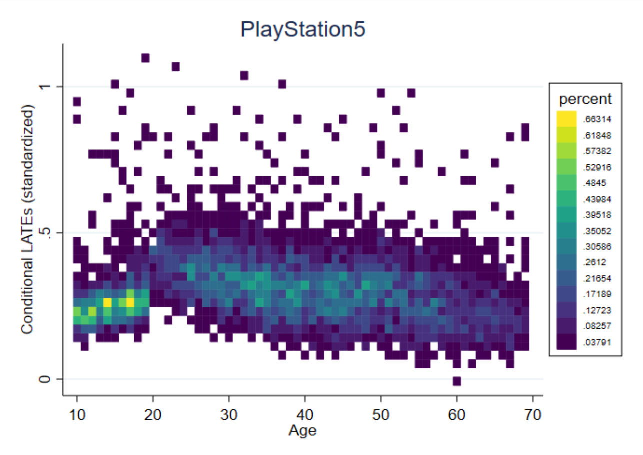

(b) PlayStation5 ownership effect by age

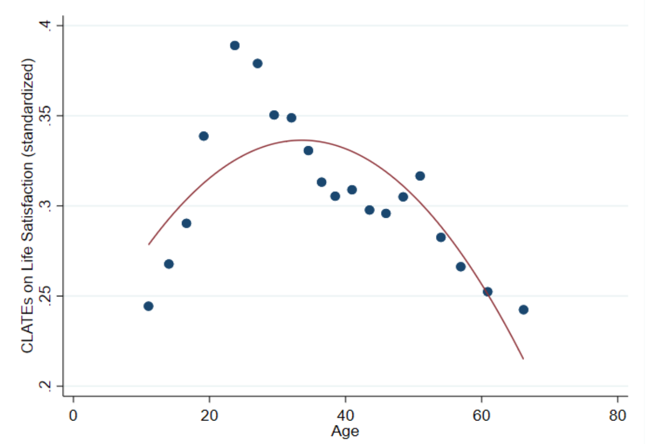

(c) PlayStation5 ownership effect by age (bin plot)

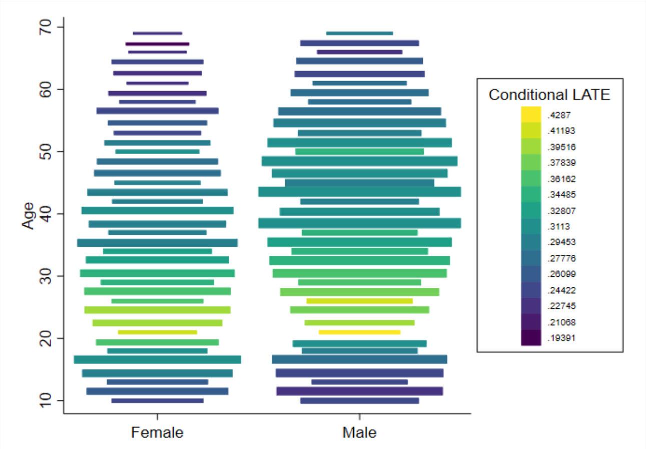

(d) PlayStation5 ownership effect by gender

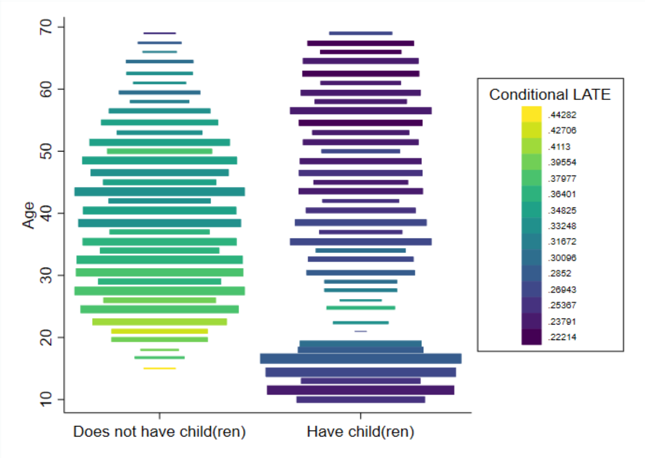

(e) PlayStation5 effect by household structure

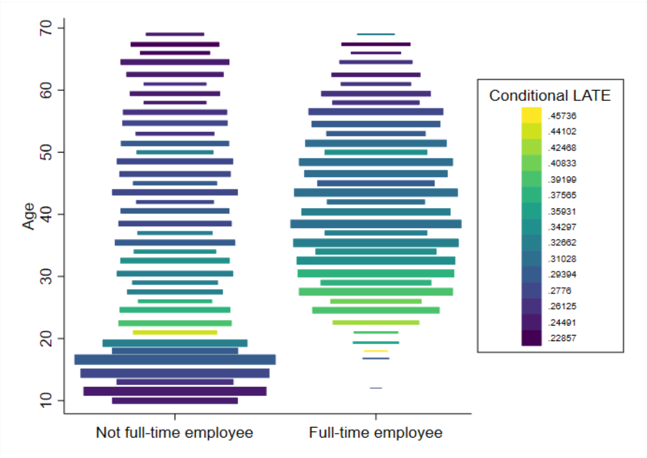

(f) PlayStation5 effect by employment status

Supplementary Figure 18: Machine learning results of effect modification in PlayStation5 ownership effect on life satisfaction (N=6,419).

Notes. CLATE, Conditional local average treatment effect; LATE, Local average treatment effect; PS5, PlayStation5; SWLS, Life satisfaction. The instrumental variable causal forest was used to estimate CLATEs on SWLS. The red vertical line present in panel a indicates LATE computed by the instrumental variable method, which uses a set of covariates common to those used in the causal forest (Supplementary Table 16). The heat plot in panel b is a two-dimensional histogram (of CLATEs) that displays the frequencies of binned values on the y-axis and x-axis variables as rectangular fields using a color gradient. A brighter color signifies a larger number of frequency occurrences in the two-dimensional histograms. Panel b portrays the magnitude of the benefit of video games on the vertical axis and age on the horizontal axis. The same estimates are depicted in the bin plot of panel

c for easy viewing. Each panel d, e, f is a heat map of a trivariate distribution where the color gradient is used to visualize the average value of z within y-axis and x-axis bins of rectangular fields. The estimated CLATEs of video game ownership on SWLS are illustrated, taking age as the y-axis and each background characteristic as the x-axis. A brighter color indicates that video game ownership is more advantageous for individuals in that particular bin. The bar width of panels d, e, and f represents the sample size for each age group. The estimates are standardized by the standard deviations.

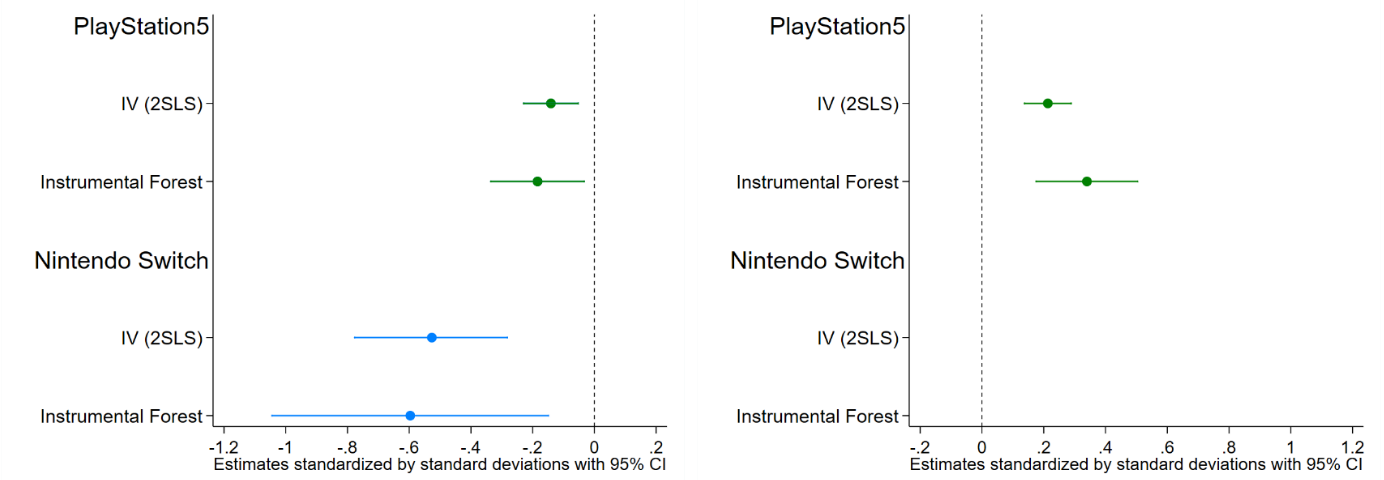

(a) Psychological distress (K6)

(b) Life satisfaction (SWLS)

Supplementary Figure 19: Comparison of estimates between instrumental variable regression and instrumental variable causal forest on the impact of video game console possession on well-being in Japan (N=8,192).

The causal effect of possession of video game consoles on well-being (LATE) is estimated by instrumental variable regression and machine learning (instrumental variable causal forest algorithm). The analysis sample is limited to those who joined game console lotteries. The point estimates (mean values) and the 95 percent confidence intervals are shown. Regression standard errors are clustered by prefectures. GRF standard errors estimated via bootstrapping. Exposure variables are possession of video game consoles (Nintendo Switch and PlayStation5 for each). The instrumental variable regression and the instrumental forests use a set of covariates shown in Supplementary Table 16. A lower K6 means having less psychological distress, while a higher SWLS means greater life satisfaction. The estimates are standardized by the standard deviations. LATE, Local Average Treatment Effect; CI, confidence intervals; IV, Instrumental Variable, 2SLS, Two-stage least squares; GRF, Generalized Random Forests.

Supplementary References.

1. Barr M, Copeland-Stewart A. Playing Video Games During the COVID-19 Pandemic and Effects on Players' Well-Being. *Games Cult*. 2022;17(1):122-139. doi:10.1177/15554120211017036

2. Halbrook YJ, O'Donnell AT, Msetfi RM. When and How Video Games Can Be Good: A Review of the Positive Effects of Video Games on Well-Being. *Perspect Psychol Sci*. 2019;14(6):1096-1104. doi:10.1177/1745691619863807

3. Granic I, Lobel A, Engels RCME. The benefits of playing video games. *Am Psychol*. 2014;69(1):66-78. doi:10.1037/a0034857

4. Hernán MA, Robins JM. *Causal Inference: What If.* Boca Raton: Chapman & Hall/CRC.; 2020.

5. Imbens GW. Matching methods in practice: Three examples. *J Hum Resour*. 2015;50(2):373-419. doi:10.3368/jhr.50.2.373

6. Austin PC. Statistical criteria for selecting the optimal number of untreated subjects matched to each treated subject when using many-to-one matching on the propensity score. *Am J Epidemiol*. 2010;172(9):1092-1097. doi:10.1093/aje/kwq224

7. Athey S, Tibshirani J, Wager S. Generalized random forests. *Ann Stat*. 2019;47(2):1179-1203. doi:10.1214/18-AOS1709

8. Athey S, Wager S. Policy Learning With Observational Data. *Econometrica*. 2021;89(1):133-161. doi:https://doi.org/10.3982/ECTA15732

9. Bertrand M, Crepon B, Marguerie A, Premand P. *Contemporaneous and Post-Program Impacts of a Public Works Program: Evidence from Côte d'Ivoire Marianne*.; 2017. http://hdl.handle.net/10986/28460

10. Iyengar R, Park YHH, Yu Q. The Impact of Subscription Programs on Customer Purchases. *J Mark Res*. 2022;59(6):1101-1119. doi:10.1177/00222437221080163

11. Jawadekar N, Kezios K, Odden MC, et al. Practical Guide to Honest Causal Forests for Identifying Heterogeneous Treatment Effects. *Am J Epidemiol*. 2023;192(7):1155-1165. doi:10.1093/aje/kwad043

12. Stekhoven DJ, Bühlmann P. Missforest-Non-parametric missing value imputation for mixed-type data. *Bioinformatics*. 2012;28(1):112-118. doi:10.1093/bioinformatics/btr597

13. Wager S, Athey S. Estimation and Inference of Heterogeneous Treatment Effects using Random Forests. *J Am Stat Assoc*. 2018;113(523):1228-1242. doi:10.1080/01621459.2017.1319839

14. Brooks JM, Chapman CG, Floyd SB, Chen BK, Thigpen CA, Kissenberth M. Assessing the ability of an instrumental variable causal forest algorithm to personalize treatment evidence using observational data: the case of early surgery for shoulder fracture. *BMC Med Res Methodol*. 2022;22(1):1-16. doi:10.1186/s12874-022-01663-0

15. Ferguson CJ. Is Psychological Research Really as Good as Medical Research ? Effect Size Comparisons Between Psychology and Medicine. *Rev Gen Psychol*. 2009;13(2):130-136. doi:10.1037/a0015103

16. Ferguson CJ. Do Angry Birds Make for Angry Children? A Meta-Analysis of Video Game Influences on Children's and Adolescents' Aggression, Mental Health,

Prosocial Behavior, and Academic Performance. *Perspect Psychol Sci*. 2015;10(5):646-666. doi:10.1177/1745691615592234

17. Johannes N, Vuorre M, Przybylski AK. Video game play is positively correlated with well-being. *R Soc Open Sci*. 2021;8. doi:10.1098/rsos.202049
18. Kelly S, Magor T, Wright A. The Pros and Cons of Online Competitive Gaming: An Evidence-Based Approach to Assessing Young Players' Well-Being. *Front Psychol*. 2021;12(651530):1-9. doi:10.3389/fpsyg.2021.651530
19. Chittaranjan Andrade M. Mean Difference, Standardized Mean Difference (SMD), and Their Use in Meta-Analysis: As Simple as It Gets. *J Clin Psychiatry*. 2020;81(5):20f13681.
20. Normand SLT, Landrum MB, Guadagnoli E, et al. Validating recommendations for coronary angiography following acute myocardial infarction in the elderly: A matched analysis using propensity scores. *J Clin Epidemiol*. 2001;54(4):387-398. doi:10.1016/S0895-4356(00)00321-8
21. Tibshirani J, Athey S, Friedberg R, et al. R Package "grf." Published online 2024. https://grf-labs.github.io/grf/
22. Robins JM, Rotnitzky A, Zhao LP. Estimation of Regression Coefficients When Some Regressors Are Not Always Observed. *J Am Stat Assoc*. 1994;89(427):846-866. doi:10.2307/2290910
23. Ryan RM, Rigby CS, Przybylski A. The motivational pull of video games: A self-determination theory approach. *Motiv Emot*. 2006;30(4):347-363. doi:10.1007/s11031-006-9051-8
24. Whitaker JL, Bushman BJ. "Remain calm. Be kind." Effects of relaxing video games on aggressive and prosocial behavior. *Soc Psychol Personal Sci*. 2012;3(1):88-92. doi:10.1177/1948550611409760
25. Primack BA, Carroll M V., McNamara M, et al. Role of video games in improving health-related outcomes: A systematic review. *Am J Prev Med*. 2012;42(6):630-638. doi:10.1016/j.amepre.2012.02.023
26. Pallavicini F, Pepe A, Mantovani F. Commercial Off-The-Shelf Video Games for Reducing Stress and Anxiety : Systematic Review. *JMIR Ment Heal*. 2021;8:1-19. doi:10.2196/28150
27. Peracchia S, Curcio G. Exposure to video games: Effects on sleep and on post-sleep cognitive abilities. A systematic review of experimental evidences. *Sleep Sci*. 2018;11(4):302-314. doi:10.5935/1984-0063.20180046
28. Suchert V, Hanewinkel R, Isensee B. Sedentary behavior and indicators of mental health in school-aged children and adolescents: A systematic review. *Prev Med (Baltim)*. 2015;76:48-57. doi:10.1016/j.ypmed.2015.03.026
29. Kowert R, Domahidi E, Festl R, Quandt T. Social gaming, lonely life? The impact of digital game play on adolescents ' social circles. *Comput Human Behav*. 2014;36:385-390. doi:10.1016/j.chb.2014.04.003
30. Yale C, Forsythe AB. Winsorized Regression. *Technometrics*. 1976;18(3):291-300. doi:10.2307/1268738
