# Supplementary material for: Causal effect of video gaming on mental well-being in Japan 2020–2022
Source: Nat Hum Behav. 2024 Aug 19;8(10):1943–56. doi: 10.1038/s41562-024-01948-y (PMC11493677; doi:10.1038/s41562-024-01948-y)
Supplement: Supplementary file 2 — Reporting Summary [file 41562_2024_1948_MOESM2_ESM.pdf]

nature portfolio

Corresponding author(s): Hiroyuki Egami

Last updated by author(s): May 30, 2024

Reporting Summary

Nature Portfolio wishes to improve the reproducibility of the work that we publish. This form provides structure for consistency and transparency in reporting. For further information on Nature Portfolio policies, see our Editorial Policies and the Editorial Policy Checklist.

Statistics

For all statistical analyses, confirm that the following items are present in the figure legend, table legend, main text, or Methods section.

n/a	Confirmed	
☐	☒	The exact sample size (*n*) for each experimental group/condition, given as a discrete number and unit of measurement
☐	☒	A statement on whether measurements were taken from distinct samples or whether the same sample was measured repeatedly
☐	☒	The statistical test(s) used AND whether they are one- or two-sided<br>*Only common tests should be described solely by name; describe more complex techniques in the Methods section.*
☐	☒	A description of all covariates tested
☐	☒	A description of any assumptions or corrections, such as tests of normality and adjustment for multiple comparisons
☐	☒	A full description of the statistical parameters including central tendency (e.g. means) or other basic estimates (e.g. regression coefficient) AND variation (e.g. standard deviation) or associated estimates of uncertainty (e.g. confidence intervals)
☐	☒	For null hypothesis testing, the test statistic (e.g. *F*, *t*, *r*) with confidence intervals, effect sizes, degrees of freedom and *P* value noted<br>*Give P values as exact values whenever suitable.*
☒	☐	For Bayesian analysis, information on the choice of priors and Markov chain Monte Carlo settings
☒	☐	For hierarchical and complex designs, identification of the appropriate level for tests and full reporting of outcomes
☐	☒	Estimates of effect sizes (e.g. Cohen's *d*, Pearson's *r*), indicating how they were calculated

Our web collection on statistics for biologists contains articles on many of the points above.

Software and code

Policy information about availability of computer code

Data collection	No specific software was used for data collection. Respondents filled out the questionnaire using their web browser.
Data analysis	STATA 16.1; R version 4.3.1; R package "grf", version 2.3.2.

For manuscripts utilizing custom algorithms or software that are central to the research but not yet described in published literature, software must be made available to editors and reviewers. We strongly encourage code deposition in a community repository (e.g. GitHub). See the Nature Portfolio guidelines for submitting code & software for further information.

Data

Policy information about availability of data

All manuscripts must include a data availability statement. This statement should provide the following information, where applicable:

- Accession codes, unique identifiers, or web links for publicly available datasets
- A description of any restrictions on data availability
- For clinical datasets or third party data, please ensure that the statement adheres to our policy

Data analyzed in this study are not openly available due to usage restrictions and licensing agreements with gameage R&I. Certain variables (i.e., video game console ownership and video gaming preference measures) are the proprietary information of gameage R&I and are not available for public dissemination. However, the data are available upon request by accredited academic researchers from the corresponding author and with permission from gameage R&I.

Research involving human participants, their data, or biological material

Policy information about studies with human participants or human data. See also policy information about sex, gender (identity/presentation), and sexual orientation and race, ethnicity and racism.

Reporting on sex and gender	Gender was considered in the heterogeneity analysis. Gender was determined based on self-reporting. Informed consent was obtained from all participants by the survey agent prior to the interview. In the case of minor participants, informed consent was taken from parents or legally authorized representatives.
Reporting on race, ethnicity, or other socially relevant groupings	N/A
Population characteristics	Japanese population aged 10-69, including half male and half female.
Recruitment	We conducted online omnibus surveys with gameage R&I, a gaming market research firm. Individuals who have pre-registered at a survey agency Cross Marketing (a pool of roughly 150,000 respondents) were sent survey offers. The stratified random sampling technique (stratified by gender, age, and gaming preference) was used. While voluntary participation in surveys can introduce self-selection bias, our comparison of respondents and non-respondents showed similar characteristics, mitigating this concern. It is acknowledged that individuals registered with the survey agency might differ from those who did not register, presenting an inherent challenge of online surveys. However, by stratifying based on gaming preference, we mitigated the concern of attracting a disproportionate number of avid gamers to our survey. Additionally, our study might have yielded context-based estimates as the data was collected during the COVID-19 period (2020-2022).
Ethics oversight	The survey was approved by the institutional review board of Takasaki City University of Economics (approval number 245-1). Informed consent was obtained from all participants by the survey agency prior to the interview. In the case of minor participants, informed consent was taken from parents or legally authorized representatives. All data was kept confidential and used only for research purposes. The study posed minimal risk to participants, and the participants' privacy was protected throughout the study. Data was anonymized to protect the participants' privacy.

Note that full information on the approval of the study protocol must also be provided in the manuscript.

Field-specific reporting

Please select the one below that is the best fit for your research. If you are not sure, read the appropriate sections before making your selection.

☐ Life sciences ☒ Behavioural & social sciences ☐ Ecological, evolutionary & environmental sciences

For a reference copy of the document with all sections, see nature.com/documents/nr-reporting-summary-flat.pdf

Behavioural & social sciences study design

All studies must disclose on these points even when the disclosure is negative.

Study description	This is a natural experimental study identifying the causal effects of video gaming on mental well-being. The study utilized a unique context where major gaming consoles, Nintendo Switch and PlayStation 5, were distributed through lotteries due to supply shortages. This setup provided a near-random variation in gaming console ownership, which we leveraged to estimate causal effects.
Research sample	Japanese population aged 10-69, including half male and half female. 21.3% were students, 10.7% were unemployed, 39.1% were full-time employees, and 57% were married. 16.0% were Hardcore gamers, 20.3% were Core gamers, 23.3% were Middle-core gamers, 17.6% were Casual gamers, and 22.9% were Non-gamers. Individuals who have pre-registered at the survey agency Cross Marketing were sampled. We intend to study the impact of video gaming on well-being, and thus, the sample is not nation-representative and rather includes more video gamers.
Sampling strategy	We conducted five rounds of omnibus online surveys with a market research firm gameage R&I. Due to the nature of the natural experiment, we did not predetermine the minimum sample size before the survey. The number of participants who entered the lottery and the number of winners were not known before the study, as these aspects were managed externally by the lottery organizers. Moreover, before conducting the study, we did not have an expectation of the effect size of winning the lottery on our outcome variables. The survey sample size (shown in the supplementary material) varied each month based on the business objective of gameage R&I, the research firm conducting the monthly survey. The survey was distributed to a large, stratified random sample of individuals pre-registered with the survey agency, ensuring representation across gender, age, and gaming preferences. This approach maximizes the likelihood of capturing a broad and representative subset of the population. The sufficiency of our sample size was assessed post hoc based on the statistical power and the precision of our estimates.
Data collection	Gameage R&I (GRI), a research firm, conducts regular monthly surveys of individuals who have pre-registered through the survey agency Cross Marketing. Their survey aims to gather consumer data specifically related to the video game industry. Additional questions of ours were incorporated throughout five surveys. Particularly, mental well-being measures (K6 and SWLS) were collected. Respondents filled out the questionnaire using their web browser. In the case of minor participants, their parents or legally authorized representatives filled out the questionnaire. [Presence of others] The data collection process was conducted online, ensuring that responses were provided in a private setting without the direct presence of researchers.[Blindness for researchers] As

the respondents answered online surveys, the researchers were initially blinded to the participants' lottery outcomes when collecting the data. However, the researchers knew whether the participants won or lost the lottery when they analyzed the data. The researchers were also aware of the study hypotheses. [Blindness for respondents] Participants were aware of whether they won the lottery or not, as this directly impacted their experience and subsequent behavior. Participants were not informed about the specific hypotheses regarding the effects of winning the lottery on mental well-being.

Timing	The survey respondents answered on 2-6 December 2020, 1-4 March 2021, 3-6 May 2021, 1-5 November 2021, and 2-7 March 2022.
Data exclusions	No data were exlcluded.
Non-participation	The survey response rate was 59.3%.
Randomization	The natural experimental study design using video game console lotteries works as randomization. We provide balance tables of a number of covariates. The multivariate regression and propensity score matching approach were used to address potential confounders.

Reporting for specific materials, systems and methods

We require information from authors about some types of materials, experimental systems and methods used in many studies. Here, indicate whether each material, system or method listed is relevant to your study. If you are not sure if a list item applies to your research, read the appropriate section before selecting a response.

Materials & experimental systems

n/a	Involved in the study
☒	☐ Antibodies
☒	☐ Eukaryotic cell lines
☒	☐ Palaeontology and archaeology
☒	☐ Animals and other organisms
☒	☐ Clinical data
☒	☐ Dual use research of concern
☒	☐ Plants

Methods

n/a	Involved in the study
☒	☐ ChIP-seq
☒	☐ Flow cytometry
☒	☐ MRI-based neuroimaging
